# Use of 3D modeling to refine predictions of canopy light utilization: A comparative study on canopy photosynthesis models with different dimensions

**DOI:** 10.3389/fpls.2022.735981

**Published:** 2022-08-18

**Authors:** Shenghao Gu, Weiliang Wen, Tianjun Xu, Xianju Lu, Zetao Yu, Xinyu Guo, Chunjiang Zhao

**Affiliations:** ^1^Information Technology Research Center, Beijing Academy of Agriculture Forestry Sciences, Beijing, China; ^2^Beijing Key Laboratory of Digital Plant, National Engineering Research Center for Information Technology in Agriculture, Beijing, China; ^3^Maize Research Center, Beijing Academy of Agriculture and Forestry Sciences, Beijing, China

**Keywords:** yield prediction, canopy photosynthesis, PAR interception, RUE, plant structure

## Abstract

Canopy photosynthesis integrates leaf functional and structural traits in space and time and correlates positively with yield formation. Many models with different levels of architectural details ranging from zero-dimensional (0D) to three-dimensional (3D) have been developed to simulate canopy light interception and photosynthesis. Based on these models, a crop growth model can be used to assess crop yield in response to genetic improvement, optimized practices, and environmental change. However, to what extent do architectural details influence light interception, photosynthetic production, and grain yield remains unknown. Here, we show that a crop growth model with high-resolution upscaling approach in space reduces the departure of predicted yield from actual yield and refines the simulation of canopy photosynthetic production. We found crop yield predictions decreased by 12.0–48.5% with increasing the resolution of light simulation, suggesting that a crop growth model without architectural details may result in a considerable departure from the actual photosynthetic production. A dramatic difference in light interception and photosynthetic production of canopy between cultivars was captured by the proposed 3D model rather than the 0D, 1D, and 2D models. Furthermore, we found that the overestimation of crop yield by the 0D model is caused by the overestimation of canopy photosynthetically active radiation (PAR) interception and the RUE and that by the 1D and 2D model is caused by the overestimated canopy photosynthesis rate that is possibly related to higher predicted PAR and fraction of sunlit leaves. Overall, this study confirms the necessity of taking detailed architecture traits into consideration when evaluating the strategies of genetic improvement and canopy configuration in improving crop yield by crop modeling.

## Highlights

- A simplified crop growth model incorporating with 3D canopy photosynthesis model can refine the predictions of light interception, photosynthetic production, and crop yield.
- *A*_*can*,_*_*t*_*, canopy photosynthesis rate at the hour *t* of the day (μmol CO_2_ m^–2^ s^–1^); *A*_*canDAY*,_*_*d*_*, daily assimilated CO_2_ by the canopy on the day *d* of year (μmol CO_2_ m^–2^ d^–1^); *A*_*i,j,t*_, instantaneous net photosynthesis rate of leaf *i* of the plant *j* within the focal area of a reconstructed canopy at the time *t* (μmol CO_2_ m^–2^ leaf s^–1^); *A*_*max*_, photosynthesis rate at saturated light conditions (μmol CO_2_ m^–2^ s^–1^); *A*_*plant*,_*_*j*_*, daily CO_2_ assimilation of the entire plant *j* (μmol CO_2_ plant^–1^ d^–1^); *Asun*_*n,a,t*_ and *Ash*_*n,t*_, instantaneous photosynthesis rate for leaves that are sunlit and shaded in the canopy layer *n* at the hour *t* of the day (μmol CO_2_ m^–2^ leaf s^–1^); CPM, canopy photosynthesis model; *DM*_*d*_, daily dry mass of canopy on the *d* day of year (g m^–2^ d^–1^); *fIPAR*, fraction of intercepted PAR; *fsun*_*n,t*_, fraction of leaves that are sunlit in the canopy layer *n* at the hour *t* of the day; *I*_*i,j,t*_, average total incident PAR intensity per leaf area for the leaf *i* of the plant *j* within the focal area of a reconstructed canopy at the time *t* of day (μmol photons m^–2^ leaf s^–1^); *I*leaf*_*n,t*_*, the total intercepted PAR per unit leaf area in the *n*th canopy layer (μmol photons m^–2^ leaf s^–1^); *IPAR*_*DAY*,_*_*d*_*, intercepted PAR by the canopy on the day *d* of year (MJ m^–2^ d^–1^); *k*, light extinction coefficient; LAI, leaf area index; *Ld* and *L*_*n*_, cumulative leaf area index at the canopy depth of *d* and at the canopy layer of *n*; RUE, radiation use efficiency during post-silking stage (g MJ^–1^); *RUE*_*DAY*,_*_*d*_*, daily radiation use efficiency (g MJ^–1^); *Y*_*p*_, predicted grain yield (Mg ha^–1^).

## Introduction

Accurate quantification of crop yield potential is a key prerequisite for improving realized yields per unit area (Lobell et al., 2009). Crop yield potential, the maximum possible regional yield for a given crop under optimal management without biotic and abiotic stresses, can be estimated by integrating the product of the daily total incoming photosynthetically active radiation (*I*_0_,*_*d*_*), fraction of intercepted photosynthetically active radiation (PAR) (*fIPAR*), radiation use efficiency (RUE), and harvest index (HI) over a growing season (Monteith, 1977; Zhu et al., 2010). Through multiplying RUE by stress-related empirical coefficients, crop yield under different levels of limitation can be obtained by crop growth models (Jones et al., 2003; Keating et al., 2003). Despite the efficiency and generality of this correction, the issue exists that RUE is a highly aggregated trait depending on cultivars, environment, and agronomic practices (Sinclair and Muchow, 1999), thus reducing the accuracy of modeling crop performance under various conditions and hampering our understanding of the photosynthetic improvement by taking potential of plant plasticity (Zhu et al., 2015). Upscaling photosynthesis from leaf to canopy has been a proven approach to overcome this limitation.

Canopy photosynthesis models (CPMs) with different organizational levels ranging from canopy-based to organ-based have been incorporated into crop growth models. The zero-dimensional (0D) CPMs consider a crop canopy as a single layer and estimate crop yield potential based on the daily intercepted PAR derived from the Beer–Lambert law (Monsi and Saeki, 1953) and RUE from the linear relationship between radiation and crop dry matter experimentally (Spitters and Schapendonk, 1990). However, the light extinction coefficient (*k*) is highly dependent on canopy architectural traits and solar elevation, and canopy photosynthesis is more sensitive to light distribution within a canopy than total light interception (Tollenaar and Dwyer, 1999). To tackle these issues, the one-dimensional (1D) CPMs consider the vertical distribution of light in canopy and used the Gaussian integration to compute canopy photosynthesis (Goudriaan, 1986). The light distribution within crop canopy is not only highly heterogeneous vertically but also horizontally in canopy, presenting a challenge to accurate the simulations of canopy photosynthesis through vertical integration in space. The two-dimensional (2D) CPMs divide the crop canopy into multiple horizontal layers that are composed of sunlit and shaded leaves and incorporate the leaf inclination distribution (Goudriaan, 1988). The 2D CPMs calculate intercepted light and leaf photosynthesis for individual layers and then integrate them over the entire canopy into canopy photosynthesis rate (Stewart et al., 2003). Furthermore, the three-dimensional (3D) CPMs calculate intercepted light for each individual leaf as a function of leaf size, angle, curvature, azimuth, etc., based on a 3D architectural canopy model coupled with a fine radiation model (Evers et al., 2010). Leaf photosynthesis rate at the organ level is then calculated and summed up to canopy photosynthesis.

The 0D, 1D, and 2D models employed the Beer–Lambert law to estimate light interception (e.g., *fIPAR*), in which the light exponential attenuation within the canopy can be expressed as a function of cumulative leaf area index (LAI) and extinction coefficient (*k*). Despite a substantial improvement in *fIPAR* over generations of genetic selection (Perez et al., 2019), the response of LAI and *k* to plant structure, plant density and planting pattern may vary in different directions (Zhang et al., 2014). A number of two canopies with different plant structure and row configuration may have the same *fIPAR* when they have the same product of LAI and *k*. This simplification, however, may overlook the difference of canopy photosynthetic gain due to the ignorance of detailed architectural characteristics such as leaf inclination, leaf azimuth, and leaf curvature in simulating light interception, and this difference has never been investigated quantitatively.

The 0D, 1D, and 2D approaches have been widely used in many crop growth models for evaluating the effects of cultivar selection, agronomic practices, and climate change on crop yield potential (van Diepen et al., 1989; Spitters and Schapendonk, 1990; Goudriaan and Van Laar, 1994; van Ittersum et al., 2003; Yang et al., 2004; Chen et al., 2013; Meng et al., 2020). Its capability of simulating light capture by heterogeneous canopies such as narrow-wide row configuration in monoculture and intercropping system is still limited. These approaches overlook the shading effects and architectural plasticity and use the average intercepted PAR by each individual layer or by an entire canopy instead, leading to an overestimation of light interception, canopy photosynthesis, and thereby crop yield. In addition, Emmel et al. (2020) found that the enhancement of crop canopy photosynthesis under the increasing fraction of diffuse radiation was primarily due to optimal light distribution within canopy *via* increasing heterogeneity of canopy architecture. Therefore, it is necessary to account for heterogeneities of light distribution in 3D space within canopy when predicting crop yield.

The light interception and photosynthetic capacity of leaves within canopy are highly heterogeneous, and the light interception estimated by a low-resolution approach has been reported to cause an overestimation of photosynthetic gain (Hammer and Wright, 1994; Zhu et al., 2012; Kim et al., 2020; Rosati et al., 2020). Spitters (1986) indicated that using light interception calculated over canopy layers led to an overestimation of canopy photosynthetic gain by up to 23% compared to multilayer approach. de Leon and Bailey (2019) found that the light interception was consistently overpredicted based on the Beer’s law compared to the 3D model in combination with a leaf-resolving radiation model, and the error became large as the plant spacing and canopy heterogeneity increased. To what extent and how do 0D, 1D, and 2D models overpredict crop yield compared to the 3D model remains unknown.

The objectives of this study were to: (1) evaluate to what extent the 3D model can further reduce the overestimations of canopy photosynthesis and crop yield; (2) to test whether the 3D model is adequate to refine simulations in light interception, canopy photosynthesis, and dry mass production; and (3) to explore underlying reasons for the overestimation of photosynthetic production using 0D, 1D, and 2D models.

## Materials and methods

### Experimental site and setup

The experiment was conducted in 2019 at the experimental station of Beijing Academy of Agriculture and Forestry Agricultural Sciences in Tongzhou (39°42′ N, 116°41′ E), Beijing, China. The soil is a brown sandy loam with an organic matter of 17.03 g kg^––1^, total soil nitrogen of 1.08 g kg^––1^, Olsen phosphorus of 0.067 g kg^––1^, and available potassium of 0.241 g kg^––1^. Maize plants of hybrid Xianyu 335 (XY335) were grown at three plant population densities: 4.5, 7.5, and 10.5 plants m^––2^ (XY4.5, XY7.5, and XY10.5). The experimental plots were laid out as a randomized block design with 3 replicates. Maize plants were sown on May 10, 2019 and harvested on October 1, 2019. Plants of the Zhengdan 958 (ZD958) at the plant density of 7.5 plants m^––2^ (ZD7.5) with a substantially different plant architecture from XY335 were selected to evaluate the difference in simulating crop performance between the 0D, 1D, 2D, and 3D models. Phenology was recorded for each plot with a 2-day interval. Silking and physiology maturity date were July 19, 2019 and September 17, 2019. Sunshine duration data were obtained from the China Meteorological Data Service Center^1^.

### Measurements

#### 3D digitalization

In total, three plants were randomly selected for each plot to collect the 3D digitalization data using a FastScan 3D digitizer (Polhemus, Colchester, VT, United States) to build 3D canopy model on 14 days after silking, after which no more architectural changes occur in maize.

#### PAR distribution within the canopy

We measured PAR with a LI-191R line quantum sensor (LI-COR Inc., Lincoln, NE, United States) from the bottom to top of the canopy with a 30-cm interval for each plot around midday on a clear day. The line sensor was placed diagonally and perpendicular to the row at each canopy height.

#### Grain yield

In total, two rows of maize plants with a total area of 12 m^2^ in the middle of each plot were harvested manually for yield determination. The total number of plant and ears were counted to determine ear density, the number of ears per m^2^ ground area, in the whole sampling area. We counted kernel number per ear for 10 randomly selected plants and then determined kernel weight by randomly selecting 1,000 kernels from these 10 plants’ subsamples. The grain yield of maize was expressed as the product of ear density, kernel number per ear, and kernel number per plot at 14% moisture content.

### Model description

**FIGURE 1:**
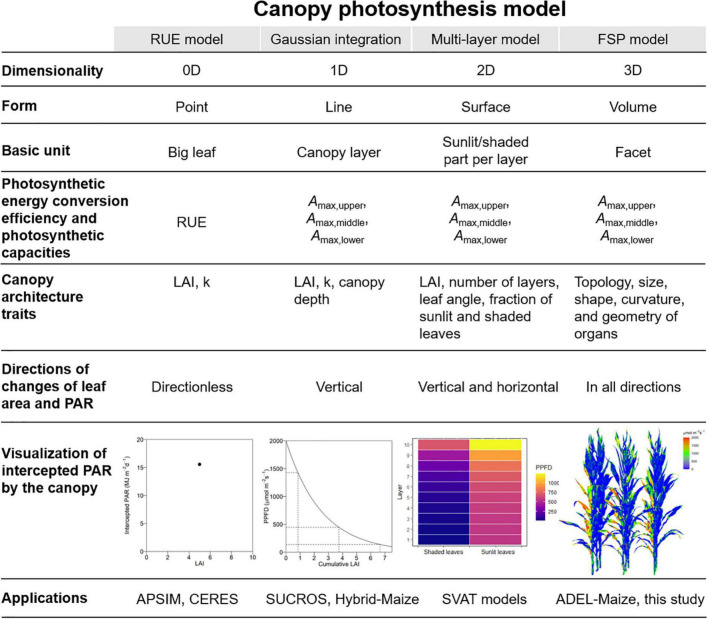
The comparison of the form, basic unit, photosynthetic energy conversion efficiency and photosynthetic capacity, canopy architecture traits, direction of canopy leaf area and PAR changes, visualization of intercepted PAR by canopy, and applications between canopy photosynthesis models with different dimensions ranging from 0D to 3D.

We extended the principle of classification of the nanostructure, which set up the dimensionality of the nanostructure as the main criterion in material science (Aversa et al., 2018), to canopy structure in CPMs. In this classification, the 0D models characterized canopy using the LAI and *k* and calculated daily PAR interception in the form of point without any direction. The 1D models characterized light attenuation in the vertical direction within the canopy and assumed that light is homogeneous horizontally. The 2D models divided the canopy into layers vertically and separated the LAI of each layer into sunlit and shaded part horizontally. The 3D models reconstructed the canopy in 3D space and calculated PAR interception on a sub-organ or a facet level in all directions. The intercepted PAR was calculated based on the big leaf, canopy layer, sunlit and shaded part per layer, and facet by the 0D, 1D, 2D, and 3D models in the form of point, line, surface, and volume (Figure 1).

Models with different dimensions ranging from 0D to 3D were generated. Each model included three main modules in common: (1) crop parameters and environmental input module for preparing plant parameters and incident PAR above canopy; (2) canopy photosynthesis module for calculating daily canopy photosynthetic production or upscaling instantaneous assimilated CO_2_ on the leaf level to daily assimilated CO_2_ per unit ground area; and (3) yield formation module for integrating daily dry mass increment to final yield. Model algorithms, calculating procedure, and variables were detailed in the Supplementary Information.

#### 3D canopy photosynthesis model

**FIGURE 2:**
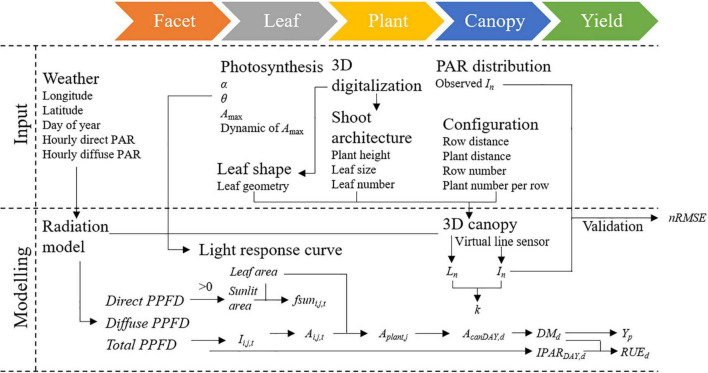
Workflow diagram of the 3D model.

Model hierarchy and calculating sequence of the 3D CPM are presented in Figure 2. The model inputs were photosynthetic parameters, 3D digitalized data, weather data including longitude, latitude, day of year, time of day, hourly direct and diffuse PAR, configuration parameters including row distance, plant distance, row number, and plant number per row. The 3D digitized data of at least three individual plants are required for the 3D canopy construction. The 3D digitized data were first used to obtain leaf shape data for constructing the leaf template database and shoot architecture data for phenotypic parameters extraction. The 3D geometric models of maize canopies were built *via* a Student’s t-distribution-based modeling approach using the 3D canopy architecture module “MaizeTypeOpt” (Wen et al., 2018, 2019). The t-distribution function was performed to generate the parameters of the traits at the scale of the individual plant and organ. The traits at the plant level are plant height and total leaf number and that at the organ level are leaf growth height, leaf inclination, azimuth angle, leaf length, and leaf width. Leaf templates in the database were then selected according to the similarity of phenotypic parameters of each leaf. After determining the configuration parameters, the 3D maize canopy was reconstructed.

**FIGURE 3:**
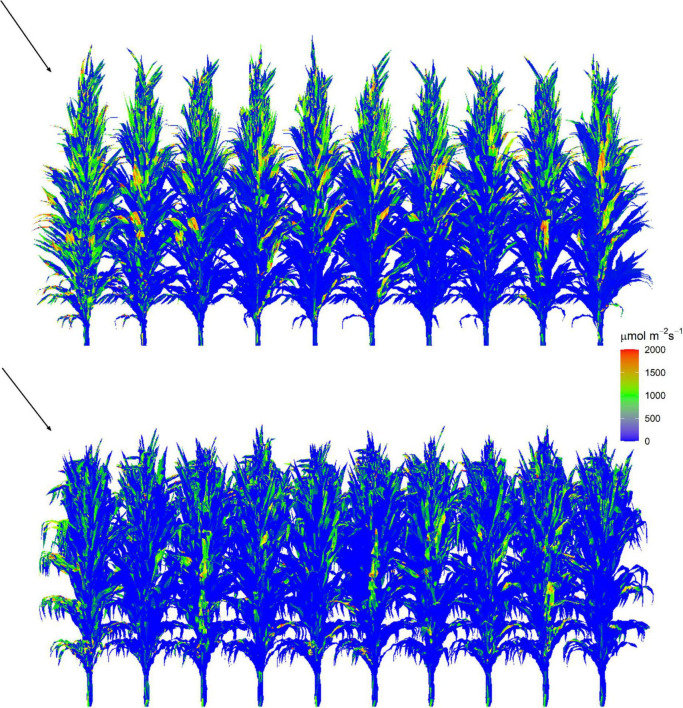
Visualization of intercepted PAR by facets within reconstructed canopy at 12:00 on a clear day on August 1, 2019 for XY335 (top) and ZD958 (bottom) at the density of 7.5 plants m^– 2^. Each canopy is composed of 200 plants with 10 rows and 20 plants per row. Red triangles represent higher values and blue triangles represent lower values. The arrow indicates the illumination direction and the sun elevation angle is 68°.

PAR distribution in each 3D maize canopy was calculated using the “Shine3DCrop,” a software for simulating radiation interception by each facet in the 3D canopy based on the ray-casting algorithm following the procedure given subsequently (Supplementary File SB and Supplementary Video). Weather data files including longitude, latitude, day of year, hourly incident PAR intensity of direct and diffuse light, and the reconstructed 3D maze canopy were set as input in this module. Canopy gap fractions (CGFs) of each canopy, which contained the probability of a diffuse beam reaching to each facet in the canopy, were calculated first (Wen et al., 2019). Then, the direct (Wang et al., 2008) and diffuse PAR distributions (Wen et al., 2019) within the canopy were calculated simultaneously at an hourly interval in separate channels. We calculated the average total incident PAR intensity per leaf area for the leaf *i* of plant *j* during the hour *t* (*I*_*i*,j,_*_*t*_*, μmol m^–2^ s^–1^) for simulating leaf photosynthesis. To reproduce the canopy microclimate and minimize border effects on light interception, simulations were run for a canopy of 10 × 20 plants, and the focal area of 2 × 2 plants was selected for calculating photosynthesis. The visualization of the 3D canopy structure and the distribution of intercepted PAR for the reconstructed canopies are shown in Figure 3. The fraction of leaves that are sunlit (*f*sun*_*i,j,t*_*) was calculated by the ratio of the total area of triangles (small facets for light calculation) that are sunlit to the total area of the corresponding leaf. Each canopy facet has a normal vector that is perpendicular to the facet surface to describe its direction. We estimated for each facet whether it is toward the sun or not during the process of calculating the direct light distribution. If the angle between the facet’s normal vector and the sun’s direction is less than 90°, the facet was determined as sunlit. Otherwise, it was shaded.

The instantaneous net photosynthesis rate of each individual leaf (*A*_*i,j,t*_) was calculated using the non-rectangular hyperbola equation (Thornley and Johnson, 1990; Supplementary Equation 3). The *A*_*i,j,t*_ of each leaf of each plant in the focal area was then summed up to obtain instantaneous assimilated CO_2_ for an individual plant (*A*_*plant*_*_,j_*, Supplementary Equation 4). The instantaneous assimilated CO_2_ per unit ground area (*A*_*can*_*_,t_*, Supplementary Equation 5) can be computed by multiplying the average *A*_*plant*_*_,j_* by plant density (Supplementary Equation 5). The daily CO_2_ assimilation of canopy (*A*_*canDAY*,_*_*d*_*) was calculated by the Supplementary Equation 6.

To evaluate the model performance in reproducing the light environment within a maize canopy, three virtual line sensors for PAR simulation in canopy were placed in the same or closely similar positions as the sensors in the experimental field (Figure 3). The 3D radiation model was further validated by comparing the simulations of PAR interception of individual leaves with that by the FastTracer, a published radiation model for 3D crop canopy (Song et al., 2013). The cumulative LAI of each canopy was calculated using the 3D canopy architecture module “MaizeTypeOpt” (Supplementary Figure 1). In this module, we divided the canopy depth into several 20-cm height intervals. We summed the area of all facets that above each canopy depth (*d*) to the cumulative leaf area. The cumulative LAI at each canopy depth (*Ld*, Supplementary Table 1) was then obtained by dividing the cumulative leaf area by the ground area of the canopy. The LAI of the canopy was equal to the cumulative LAI at the bottom (Supplementary Figure 1). The individual leaf area was calculated by the total area of all facets that belong to this leaf. By summing up all the individual leaf area for an individual plant, the leaf area per plant was calculated.

#### 2D canopy photosynthesis model

The 2D model was developed based on the multilayer model for maize by Bonelli and Andrade (2020) with incorporating specified leaf inclination distribution instead of using the spherical distribution. In total, nine equally spaced classes of leaf inclination from 0 to 90° were divided, and different canopy layers were assumed to hold the same leaf inclination distribution (Supplementary Table 2). The canopy was divided into ten equally spaced layers from canopy top with a cumulative LAI (*L*_*n*_) of 0 to bottom with that of LAI, each of which was divided into sunlit leaves that received both direct and diffuse light and shaded leaves that only received diffuse light. Instantaneous leaf photosynthesis rate was calculated by the non-rectangular hyperbola equation for sunlit (*Asun*_*n,a,t*_) and shaded part (*Ash*_*n,t*_) separately in each layer (Thornley and Johnson, 1990; Supplementary Equations 20, 21) and then integrated to the entire canopy after taking leaf inclination distribution (*f*_*a*_, Supplementary Table 2) and fraction of sunlit leaves (*fsun*_*n,t*_, Supplementary Equation 17) into consideration (*A*_*can*,_*_*t*_*, Supplementary Equation 25). The *A*_*can*,_*_*t*_* was integrated over daytime to obtain the daily assimilated CO_2_ for canopy (*A*_*canDAY*,_*_*d*_*, Supplementary Equation 6).

#### 1D canopy photosynthesis model

The 1D model was built based on the approach to upscaling photosynthesis on the leaf level to that on the canopy level by the Hybrid-Maize (Yang et al., 2004). This model assumed that the light attenuation along the canopy depth follows the Beer’ rule, and the canopy is homogeneous horizontally. The three-point Gaussian integration that includes a depth-dependent leaf photosynthetic capacity (*A*_*max*_, Supplementary Table 3) was applied for the spatial integration to canopy photosynthesis. The photosynthesis rate was calculated in the middle, at a relative distance of $\sqrt{0.15}$LAI from the upper and lower sides of the middle (Supplementary Equations 26–28). The weights of photosynthesis rate at these three points are 0.277778, 0.444444, and 0.277778 when upscaling them to canopy photosynthesis (Supplementary Equation 35). The *A*_*can*,_*_*t*_* was integrated over daytime to obtain the daily assimilated CO_2_ for canopy (*A*_*canDAY*,_*_*d*_*, Supplementary Equation 6).

#### 0D canopy photosynthesis model

The 0D model was developed based on the LINTUL crop growth model (Spitters and Schapendonk, 1990) in which daily dry mass production was calculated by multiplying daily total incoming PAR, by *fIPAR*, and by RUE (Supplementary Equations 37–39). The RUE for XY335 and ZD958 was collected from the literature and assumed to be same and constant (Supplementary Table 1).

#### Estimation of light extinction coefficient

The light extinction coefficient of 1D and 0D model was obtained by performing non-linear fitting of negative exponential equation on simulated PAR vertical distribution along cumulative LAI to minimize the perturbation caused by the error of light measurements (Wiechers et al., 2011) and of mismatch with the aforementioned 3D model. The light attenuation in relation to the canopy depth was approximated by the Beer’s Law equation:


(1) $$I_{L\,d}=I_{0}\,e^{-k\,\,L\,d}$$

where *I*_0_ and *I*_*Ld*_ (μmol m^–2^ s^–1^) are the incident PAR above and at the canopy depth of *n* with a corresponding cumulative LAI of *Ld*, and *k* is the light extinction coefficient.

#### Yield formation module

To consider the respiration of photosynthetic and non-photosynthetic organs, we converted the *A*_*canDAY*,_*_*d*_* to canopy dry mass increment (*DM*_*d*_) using a conversion ratio (*C*_*r*_, Supplementary Equation 40), taking biochemical conversion and maintenance respiration into consideration (Wu et al., 2018). The *DM*_*d*_ was then divided by daily intercepted PAR (*IPAR*_*DAY,d*_, Supplementary Equation 8 for 3D, and Supplementary Equation 17 for 2D and 1D models) to yield daily RUE (*RUE*_*DAY*,_*_*d*_*, Supplementary Equation 41). Based on the evidence that carbohydrates in grain are almost fixed by leaves during the post-silking stage (Lee and Tollenaar, 2007), we assumed that the final grain yield is equal to the post-silking dry mass accumulation in maize. Therefore, the predicted yield (*Y*_*p*_, Mg ha^–1^) was calculated as the sum of *DM*_*d*_ from silking to maturity (Supplementary Equation 42).

### Vertical distribution and temporal dynamics of photosynthetic capacity

Leaf photosynthetic light response curves (*A*-Q curve) of leaves at ranks 10, 15, and 20 for XY335 at the density of 7.5 plants m^–2^ were measured on 20 July 2019 using a portable photosynthesis system (LI-6400, LI-COR, Lincoln, NE, United States). The PAR intensity was set to 0, 50, 100, 150, 200, 250, 300, 400, 500, 600, 700, 800, 1,000, 1,200, 1,400, and 1,600 μmol photons m^2^ s^–1^. The non-rectangular hyperbola is more straightforward and versatile in expression with parameters containing underlying biological meaning and can also produce satisfactory fit to a wide range of leaf photosynthesis data (Thornley and Johnson, 1990). We estimated the apparent quantum yield (α), empirical curvature coefficient (θ), and light-statured photosynthesis rate (*A*_*max*_) by a non-linear least squares function and predicted instantaneous leaf photosynthesis rate by the parametrized non-rectangular hyperbola equation. To avoid perturbations of light response curve, we assumed that the α and θ are homogeneous across canopy and constant over grain-filling stage and also used the non-rectangular hyperbola for the light response curve in the 3D, 2D, and 1D models (Supplementary Equations 3, 20–21, 32–34). The empirical relationships between *A*_*max*_ for individual leaves at different phytomer ranks (leaf number counting from the plant base) and days after silking (DAS) were used to describe the spatial-temporal pattern photosynthetic capacity (Supplementary Figure 2). We collected the *A*_*max*_ at different leaf positions and its temporal dynamics during post-silking period for the cultivar XY335 from the work of Chen et al. (2016). To avoid the variations caused by photosynthetic energy conversion efficiency or photosynthetic capacity, we assumed that the ZD958 have the same photosynthetic energy conversion efficiency as XY335 which was confirmed previously by their similar RUE (Zhao et al., 2015) and that have the same leaf photosynthetic capacity (*A*_*max*_) as XY335. For computing efficiency and consistency, we used layers 1–3, 4–7, and 8–10 in the 2D model and phytomers 16–20, 12–15, and 7–11 in the 3D model to represent upper, middle, and lower canopies for describing the spatial heterogeneity of photosynthetic capacity (Supplementary Table 3).

### Model validation

The goodness of fit between simulation and observation was evaluated by the coefficient of determination (*r*^2^) and the normalized root mean square error (*nRMSE*, defined as RMSE normalized by the mean value of observations). The model performance is considered excellent with nRMSE < 10%, good if 10–20%, acceptable if 20–30% and poor if > 30% (Jamieson et al., 1991).

### Simulation scenarios

Simulations were run using the same crop management and environmental factors as the field experiment. A number of three replicate canopies of each treatment were generated for simulating light distribution using the 3D model. Simulation scenarios using the 0D model with a constant RUE and using the 1D, 2D, and 3D models with and without considering spatial-temporal pattern of photosynthetic capacity were formulated for XY335 at three densities (Supplementary Table 4). To characterize the crop performance for two cultivars with different plant architectures at the same density when canopy closure occurs, the 0D model with a constant photosynthetic energy conversion efficiency and the 1D, 2D, and 3D models with considering spatial-temporal pattern of photosynthetic capacity were used.

### Statistical analysis

Data analysis and visualization were performed with R language version 4.0.5 (R Core Team, 2021). The means of the results for each treatment were compared by the least significant difference (LSD test at the probability of 0.05) using the “agricolae” package of R.

## Results

### Validation of 3D model performance in simulating light distribution

**FIGURE 4:**
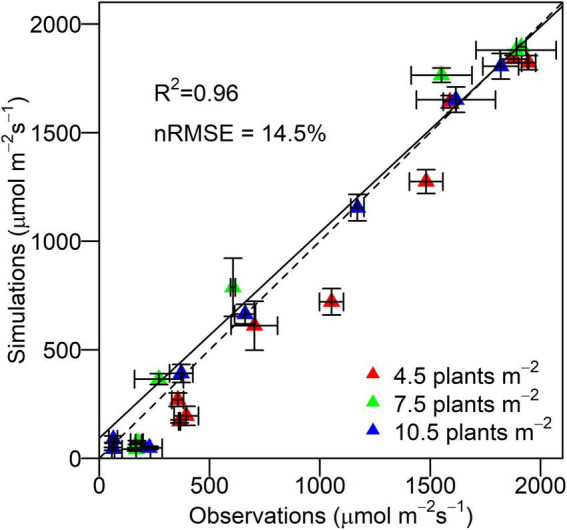
Observations of irradiant PAR by quantum sensors and simulations of irradiant PAR by virtual line sensors in the 3D model at different canopy depths at the density of 4.5, 7.5, and 10.5 plants m^– 2^ for XY335. Error bars indicate the SE. The dashed line is the 1:1 line. The solid line is the regression line.

The 3D model was evaluated by comparing simulations with observations of PAR at different canopy depths at different densities. The simulated PAR at different layers of canopy was quantitatively in line with the observations with an overall *r*^2^ of 0.96 and *nRMSE* of 14.5%, demonstrating the capability of the proposed 3D maize model in reproducing the canopy light environment across densities (Figure 4). The discrepancy between simulations and observations increased at lower plant population densities (*nRMSE* of 15.8% at 4.5 plants m^–2^, *nRMSE* of 14.8% at 7.5 plants m^–2^, and *nRMSE* of 8.8% at 10.5 plants m^–2^). The PAR interception by individual leaves simulated by the Shine3DCrops was qualitatively in agreement with that by the FastTracer, a published 3D radiation model (*RMSE* of 36.3 μmol m^–2^ s^–1^ and R^2^ of 0.57) (Supplementary Figure 3).

### Architectural traits, light extinction coefficient, and fraction of intercepted PAR of different canopies

The plant architectural traits were significantly affected by cultivar and plant density (Table 1). There was no significant difference in plant height and leaf number between plant densities for XY335. The total leaf area per plant decreased, but the LAI increased significantly with increasing density. The *fIPAR* increased with increasing plant density and reached the maximal of 0.99 at 10.5 plants m^–2^, suggesting a complete canopy closure. Despite substantial differences in architectural traits at the plant and canopy level between XY7.5 and ZD7.5, their *fIPAR* were similar.

**TABLE 1:** Phenotypic traits, light extinction coefficient and fraction of intercepted PAR (f*IPAR*) of maize canopy for different treatments.

Treatment	Plant height (cm)	Leaf number	Leaf area per plant (cm^2^)	LAI	*k*	*fIPAR*
XY4.5	296.4 ± 3.43a	20.6 ± 0.25ab	9236.9 ± 313.5a	4.16 ± 0.14c	0.72	0.95
XY7.5	299.1 ± 9.16a	20.8 ± 0.48ab	8421.7 ± 397.3a	6.32 ± 0.30b	0.62	0.98
XY10.5	298.6 ± 9.67a	20.0 ± 0.00b	6977.6 ± 267.9b	7.33 ± 0.28a	0.70	0.99
ZD7.5	255.7 ± 5.63b	21.0 ± 0.00a	9281.4 ± 235.2a	6.96 ± 0.18ab	0.49	0.97

The absence of shared letters between treatments indicates the significant difference at the level of 0.05.

### Simulated maize yield using the 0D, 1D, 2D, and 3D models

**FIGURE 5:**
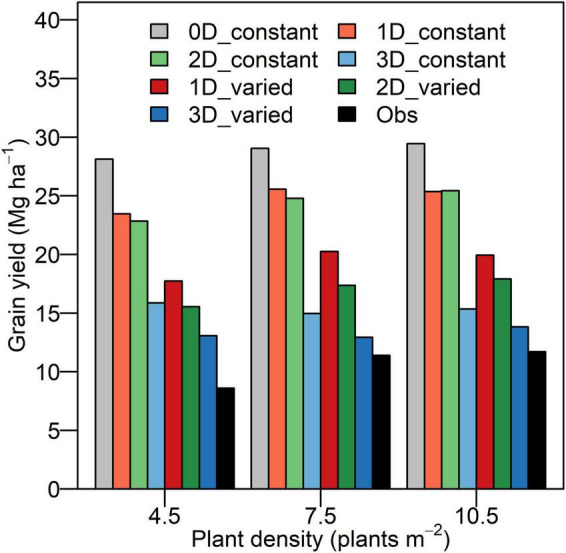
Simulated grain yield potential and experimental yield at three densities (4.5, 7.5, and 10.5 plants m**^–^**^2^) for XY335. The 0D_constant represents the 0D model with a constant RUE. The 1D_constant, 2D_constant, and 3D_constant represent the 1D, 2D, and 3D models with uniform and constant photosynthetic capacity. The 1D_varied, 2D_varied, and 3D_varied represent the 1D, 2D, and 3D models with heterogeneous and varied photosynthetic capacity. The Obs represents the experimental yield.

The discrepancy between *Y*_*p*_ and the experimental yield was reduced from the 0D to 3D models across densities (Figure 5). The *Y*_*p*_ reached the maximal of 28.1, 29.1, and 29.4 Mg ha^–1^ using the 0D model and reached the minimum of 13.1, 12.9, and 13.8 Mg ha^–1^ using the 3D model with heterogeneous and varied photosynthetic capacity at density of 4.5, 7.5, and 10.5 plants m^–2^, respectively. In comparison with the *Y*_*p*_ by the 0D model, *Y*_*p*_ decreased by 16.6, 12.0, and 13.9% using the 1D model, by 18.8, 14.7, and 13.6% using the 2D model, and by 43.6, 48.5, and 47.9% using the 3D model with uniform and constant photosynthetic capacity over the entire canopy at the density of 4.5, 7.5, and 10.5 plants m^–2^. Under the scenario with heterogeneous and varied photosynthetic capacity, the *Y*_*p*_ decreased by 12.3, 14.2, and 10.2% using the 2D model and by 26.2, 36.1, and 30.7% using the 3D model compared to that by the 1D model at three densities. The *Y*_*p*_ simulated by the 3D model was the closest to experimental yield, and the discrepancy in maize decreased at higher densities (Figure 5).

### Light interception, dry mass production, and canopy photosynthesis for two closed canopies with different plant architecture using the 0D, 1D, 2D, and 3D models

**FIGURE 6:**
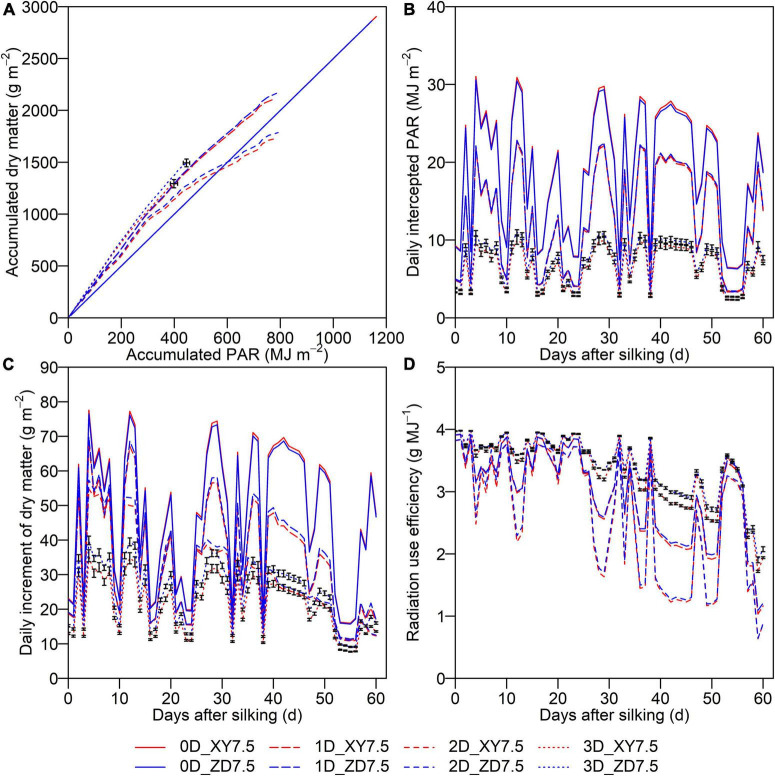
Cumulative dry matter after silking as a function of accumulated PAR **(A)** and dynamics of daily intercepted PAR **(B)**, daily increment of dry matter **(C)**, and daily radiation use efficiency **(D)** during post-silking period. Solid lines denote the 0D model, longdash lines denote the 1D model, dashed lines denote the 2D model, and dotted lines denote the 3D model. Blue denotes ZD958 and red denotes XY335 at the density of 7.5 plants m**^–^**^2^. Error bars indicate the SE.

The accumulated PAR interception and dry matter over the post-silking period simulated by the 3D model for two cultivars at the density of 7.5 plants m^–2^ were significantly different (*p* < 0.05), whereas the difference was slight by the 0D, 1D, and 2D models (Figure 6). The accumulated PAR for XY335 and ZD958 was 1,162.1 and 1,145.5 MJ m^–2^ by the 0D model, whereas that decreased to 797.3 and 785.9 MJ m^–2^ by the 1D model, to 788.7 and 791.4 MJ m^–2^ by the 2D model, and further to 398.9 and 445.9 MJ m^–2^ by the 3D model, showing a decreasing trend with increasing the resolution of light simulation. The accumulated dry matter after silking for XY335 and ZD958 was 2,905 and 2,864 g m^–2^ by the 0D model, 2,024 and 2,166 g m^–2^ by the 1D model, 1,736 and 1,787 g m^–2^ by the 2D model, and 1,293 and 1,494 g m^–2^ by the 3D model (Figure 6A). There was a significant difference on *IPAR*_*DAY*,_*_*d*_* simulated by the 3D model between XY335 and ZD958, whereas the difference was slight when using the 0D, 1D, and 2D models. The *IPAR*_*DAY*,_*_*d*_* by the 0D, 1D, and 2D models was increased by 129.2–212.7, 24.6–134.1, and 28.0–129.6% in comparison with the 3D model (Figure 6B). The discrepancy in the *IPAR*_*DAY*,_*_*d*_* between the 0D, 1D, 2D, and 3D models sharply increased on the days with higher *IPAR*_*DAY*,_*_*d*_* (Figure 6B), thereby leading to a similar departure in the *DM*_*d*_ (Figure 6C). The *DM*_*d*_ and *RUE*_*DAY*,_*_*d*_* by the 1D, 2D, and 3D models showed a characteristic decreasing pattern over the post-silking period (Figures 6C,D), whereas the 1D and 2D models produced larger variations than the 3D model due to dramatic fluctuations of simulated *IPAR*_*DAY*,_*_*d*_* (Figure 6B). Moreover, the difference in *RUE*_*DAY*,_*_*d*_* between those two canopies simulated by the 3D model was aggravated during the later grain-filling period (Figure 6D).

**FIGURE 7:**
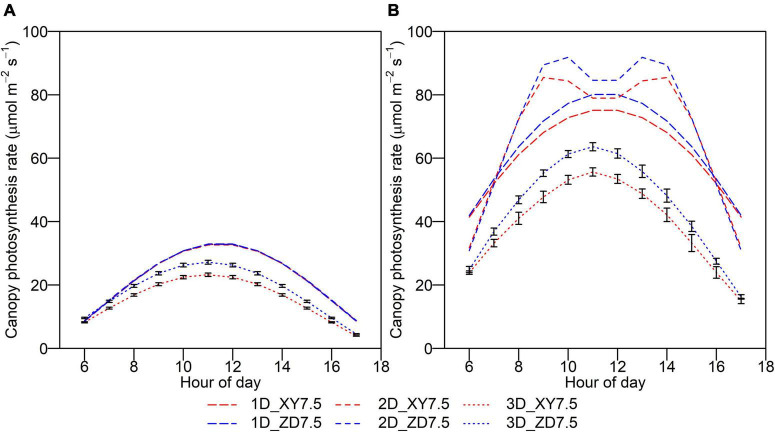
Daily course of instantaneous canopy photosynthesis rate for ZD958 (blue) and for XY335 at the density of 7.5 plant m**^–^**^2^ (red) on an overcast day **(A)** and a clear day **(B)** by the 1D (longdash lines), 2D (dashed lines), and 3D models (dotted lines). Error bars indicate the SE.

The *A*_*can*_*_,t_* showed a diurnal pattern which peaked around midday, with an increase before and a decrease after (Figure 7). The *A*_*can*_*_,t_* of XY335 simulated by the 3D model was significantly lower than that of ZD958, and the difference diminished toward sunrise and sunset, whereas this difference was not captured by the 1D and 2D models on an overcast day. The *A*_*can*_*_,t_* simulated by the 1D and 2D models were much higher than that by the 3D model with an overall increase of 50.3 and 53.7%, respectively.

### Intercepted PAR by leaves and fraction of sunlit leaves simulated by the 2D and 3D models

**FIGURE 8:**
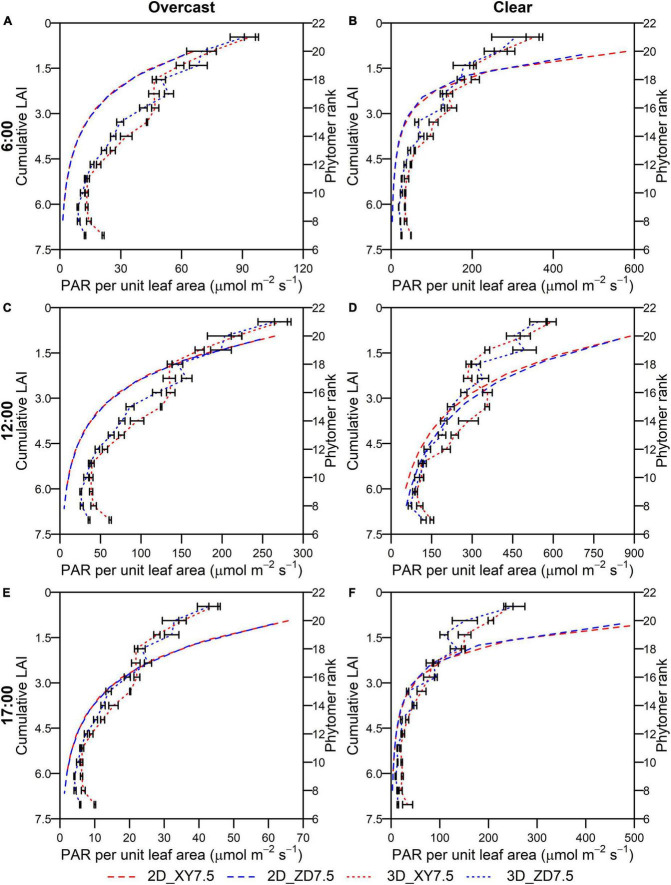
Intercepted PAR per unit leaf area at different cumulative LAI by the 2D model (dashed in left *y*-axis) and ranks by the 3D model (dotted in the right *y*-axis) over canopy at 6:00 **(A,B)**, 12:00 **(C,D)**, and 17:00 **(E,F)** for ZD958 (blue) and XY335 (red) at the density of 7.5 plant m**^–^**^2^ on an overcast day **(A,C,E)** and a clear day **(B,D,F)**. Error bars indicate the SE.

The intercepted PAR per unit leaf area for the XY335 and ZD958 at the density of 7.5 plants m^–2^ simulated by the 2D (*I*leaf*_*n,t*_*) and 3D model (*I*_*i,j,t*_) showed a consistent decline toward the bottom of the canopy irrespective of weather conditions (overcast and clear) and solar elevations (Figure 8). There was no difference in *I*leaf*_*n,t*_* across layers between the XY335 and ZD958 on either overcast or clear days (Figure 8). The *I*_*i,j,t*_ for the XY335 was significantly higher than that for the ZD958 at most phytomers irrespective of solar elevations with an overall increase of 20.2 and of 33.4% on the overcast and the clear day (Figure 8). The *I*_*i,j,t*_ at higher ranks of the ZD958 was significantly greater than that of the XY335 on the overcast day (Figures 8A,C,E) and at noon on the clear day (Figure 8D) (*p* < 0.05). The *I*leaf*_*n,t*_* were qualitatively in line with the *I*_*i,j,t*_, whereas the departure increased under clear conditions especially at the upper layers of canopy (Figure 8).

**FIGURE 9:**
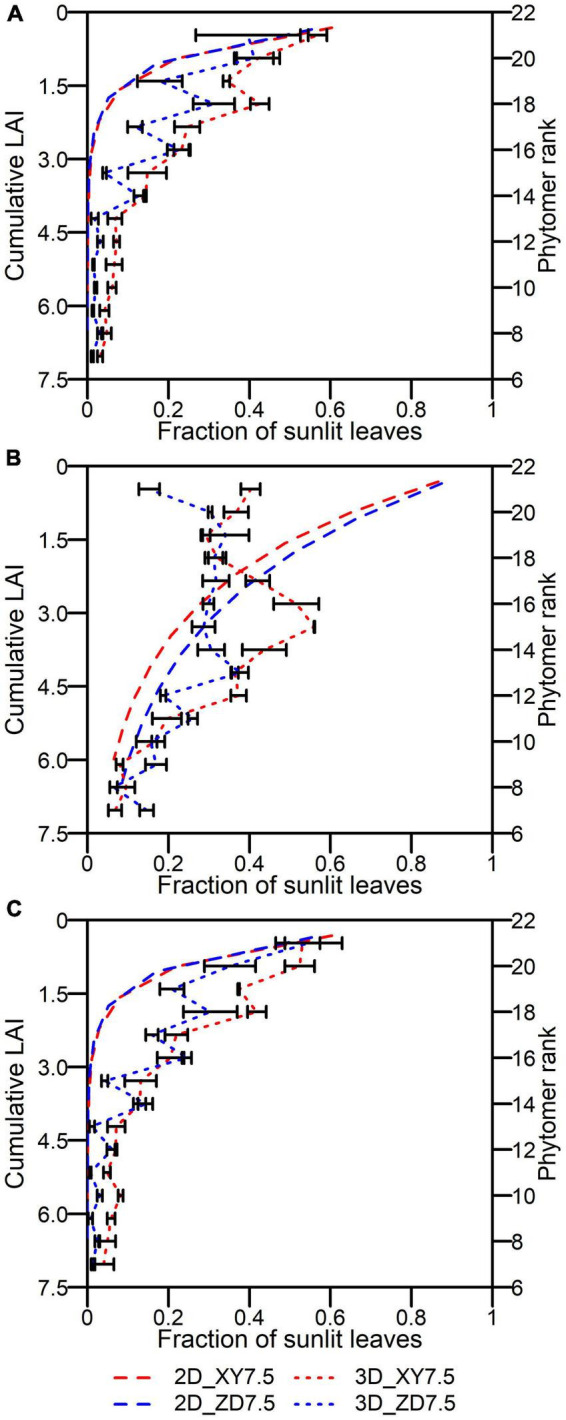
Fraction of leaves that are sunlit at different layers by the 2D model (dashed in the left *y*-axis) and ranks by the 3D model (dotted in the right *y*-axis) over canopy at 6:00 **(A)**, 12:00 **(B)**, and 17:00 **(C)** for ZD958 (blue) and for XY335 (red) at the density of 7.5 plants m**^–^**^2^ on an overcast day. Error bars indicate the SE.

The fraction of leaves that are sunlit decreased downwardly in canopy by both the 2D and 3D models (Figure 9). There was no difference in *fsun*_*n,t*_ at sunrise and sunset between XY335 and ZD958 by the 2D model. The *fsun*_*i,j,t*_ of XY335 was significantly higher than that of ZD958 across phytomer ranks at a very low solar elevation by the 3D model (Figures 9A,C). The *fsun*_*i,j,t*_ by the 3D model fluctuated in the upper canopy and reached the maximal of 0.56 at rank 15 for XY335 and of 0.38 at rank 13 for ZD958, after which the *fsun*_*i,j,t*_ decreased toward plant base at 12:00 (Figure 9B). The *fsun*_*n,t*_ by the 2D model was lower than the *fsun*_*i,j,t*_ by the 3D model over canopy at sunrise and sunset (Figures 9A,C). Despite the qualitative agreement between *fsun*_*n,t*_ and *fsun*_*i,j,t*_ in lower canopies across cultivars, the discrepancy increased and *fsun*_*n,t*_ was much higher than *fsun*_*i,j,t*_ in upper canopies at noon (Figure 9B).

## Discussion and conclusion

The proposed 3D model enables accurate simulation of canopy light distribution despite a slight underestimation at lower densities. By comparing the model performance with incorporating 0D, 1D, 2D, and 3D canopy photosynthesis models in simulating maize photosynthetic production, we quantified the effects of upscaling approaches with higher spatial resolution on predicting grain yield in maize. Overall, the predicted maize yield increased with increasing the degree of simplification of canopy photosynthesis model that was determined by both the light simulation and upscaling approach, suggesting that crop growth model based on a 0D, 1D, and 2D approaches may result in a considerable departure from the actual photosynthetic production. Moreover, our results suggest that a dramatic discrepancy in light interception between two closed canopies with different plant structures occurs from the view of 3D model, thus necessitating the consideration of 3D architectural traits in understanding the physiological processes driven by local light condition. The overestimation of light interception by leaves in the upper canopy was found to be the possible reason for the overestimation of grain yield. Finally, the proposed 3D model provides an instrumental tool in characterizing the canopy light environment and facilitating accurate and high-throughput screening for genotypes with higher RUE.

### Evaluation of 3D model performance

The 3D model performed well in profiling canopy light distribution, estimating grain yield, and characterizing the decreasing pattern of daily RUE as an emergent property for a range of canopy architectures. First, despite the slight underestimation of PAR interception at lower canopy at lower density (Figure 4), the 3D model reproduced accurately the light distribution on a clear day across plant densities in maize. This satisfactory agreement for the XY335 at three densities at a different experimental location on an overcast day was further confirmed by Wen et al. (2019). Second, the crop yield potential of XY335 at 6 plants m^–2^ can be achieved under the similar condition and optimal management (12.1 Mg ha^–2^, Chen et al., 2016), demonstrating the capability of our 3D model in simulating maize grain yield without including any other modifiers. Third, RUE at silking stage simulated by our 3D model was consistent with the potential RUE of 4 g MJ^–1^ in APSIM-Maize (Brown et al., 2014) and a multilayer model for maize by Bonelli et al. (2020), after which a progressively decreasing pattern toward maturity was observed by our proposed 3D model.

Radiation use efficiency is not only highly dependent on cultivars, environments, and managements, but also sensitive to crop status over the growing season. However, the inability to capture RUE variation between cultivars and environments presents an increasing challenge for simulating photosynthetic production using the 0D models (Wang et al., 2019). In addition, several 0D models (e.g., APSIM and CERES) simulated photosynthetic production using a constant RUE modified by stress factors over growing season (Jones et al., 2003; Keating et al., 2003). Such approach is, however, at odds with the known seasonal variation in RUE (Sinclair and Muchow, 1999). The 1D and 2D models characterized the decreasing pattern of daily RUE as the result of the decrease in leaf photosynthetic capacity, which was in line with the simulation study of Wu et al. (2018); Bonelli and Andrade (2020). These two models, however, were still limited in capturing the difference between cultivars with contrasting plant architectures (Figure 6D). The limitation was overcome by a straightforward and precise upscaling approach from the leaf photosynthesis on the organ level to the canopy photosynthetic production on the canopy level. The RUE on average over post-silking stage by the 3D model was 3.35 and 3.43 g MJ^–1^ for XY335 and ZD958 at the density of 7.5 plants m^–2^, which were higher than that by 0D, 1D, and 2D models (Figure 6D). This is because the intercepted PAR by a canopy was overestimated due to the absence of considering shaded leaves in detail (Figure 6B). The cultivar-specific decreasing pattern of daily RUE predicted by the 3D model can be set as the key parameter to 0D models such as the APSIM and CERES.

### The discrepancy between 0D, 1D, 2D, and 3D models in simulating photosynthetic production

The *Y*_*p*_ by the 1D, 2D, and 3D models was reduced by 12.0–16.6, 13.6–18.8, and 43.6–48.5% in comparison with that by the 0D model (Figure 5). This reduction is mainly caused by the different approaches to simulating PAR interception and to upscaling from leaf to canopy. Although the *I*_*i,j,t*_ simulated by the 3D model followed a similar pattern of attenuation to the *I*leaf*_*n,t*_* on each individual layer by the 2D model (Figure 8), the former was much lower than the latter in the upper canopy on a clear day (Figure 3). Because erect leaves in the upper canopy are in parallel with the direct light at midday, thereby reducing the light interception by the leaf surface (Figure 2). This result was qualitatively in line with the study by de Leon and Bailey (2019), suggesting an overestimation of total daily absorbed radiation by up to 21% and a larger overestimation near midday may be due to the simplified approach to obtaining parameters when using the Beer’s law. A markedly increasing overestimation of canopy photosynthesis under higher atmospheric transmission was also identified by Spitters (1986). Another reason for this overestimation is the convex shape of photosynthesis light response curve (Spitters, 1986; De Pury and Farquhar, 1997). In light response curves, the photosynthesis rate of shade leaves linearly correlates with incident PAR, while that of leaves in sunflecks is mostly under saturation (Yin and Struik, 2015). This results in an overestimation of canopy photosynthesis by spatial integration with instantaneous photosynthesis driven by the averaged PAR over canopy (De Pury and Farquhar, 1997). Moreover, the overestimated fraction of sunlit area in the upper canopy at higher solar elevation (Figure 9) may be another reason for the discrepancy between the 2D and 3D models.

The 3D model predicts reasonable maize yield potential without any parameterization for empirical stress-related coefficients. The deviation of *Y*_*p*_ from experimental yield increased at lower densities may be attributed to the absence of sink strength in determining the yield formation in the current models of this study. This simplification did not reproduce the quadratic relationship between yield and density (Luo et al., 2020). Soil fertility, irrigation, and pesticide were mostly well managed in experimental trials at different population densities, allowing the intraspecific competition for light to be the major factor in limiting yield improvement. The 3D model that simulates the light interception at the facet level fully accounted for the competition and self-shading, and canopy photosynthetic production would be as close as possible to attainable yield as an emergent consequence without any modifiers for stresses.

### The substantial difference in canopy photosynthetic production between cultivars simulated by high-resolution models

We dissected the substantial difference in photosynthetic production between two canopies with similar *fIPAR* (Figure 6). The *fIPAR*, expressed as the function of LAI and *k*, aggregates the effects of leaf inclination, leaf size, leaf shape, leaf azimuth, and even the internode length. Although efforts have been made to quantify the relationship between *k* and row space (Flénet et al., 1996), LAI and *k* failed to identify the effects of plant architecture, canopy configuration, and their combinations on light intercepted by leaves. Accumulated intercepted PAR over post-silking stage simulated by the 3D model for the ZD7.5 was 11.8% greater than the XY7.5 (Figure 6). Leaves of ZD958 were bigger and more curved than that of XY335 (Figure 3 and Table 1) in the upper canopy, allowing less PAR penetration into the lower canopy and less *I*_*i,j,t*_ in the middle and lower canopy (Figure 8). In addition, leaves that are perpendicular to the direction of direct light and in open space intercepted more PAR, and leaves that are parallel to the direction of direct light and under heavy shading intercepted less (Figure 3). Increased *I*_*i,j,t*_ by the more erect plants of XY7.5 compared to ZD7.5 was evidenced in Goudriaan (2016), suggesting that the light interception by erect leaves may largely exceed that of a horizontal surface for low solar elevations. The increased *I*_*i,j,t*_, however, was totally offset by the decreased individual leaf area (Table 1), resulting in an increase of 4.1–17.0% in *A*_*can*,_*_*t*_* and of 15.5% in *Y*_*p*_ for ZD958 in comparison with that for XY335 at the density of 7.5 plants m^–2^ (Figures 6A, 7). This result is not consistent with the simulations for cultivars with different plant architectures but with the same LAI by Tollenaar and Dwyer (1999); Lee and Tollenaar (2007), which indicated that a more erect plant produced 14–30% increase in canopy photosynthesis and an approximately 15% increase in dry mass accumulation over growing season for maize.

### Designing optimal planting pattern and plant architecture

Crop yield potential has been mostly evaluated by crop growth model incorporating 0D, 1D, and 2D canopy photosynthesis models. The assumption of this approach is, however, at odds with either the uneven distribution of leaf inclination at the plant level or the heterogeneous canopy structure at the canopy level. In a narrow-wide row planting pattern, leaves in the wide rows are possibly different than that in the narrow ones due to the plastic response (Zhu et al., 2015), leading to a more heterogeneous light distribution within the canopy. In addition, quantitative trait locus (QTL) regulating leaf angle has been identified at the level of phytomers (Tian et al., 2019), suggesting that designing maize ideotype with non-uniform leaf angle distribution specified for individual leaves become feasible. The 2D model would no longer be appropriate to calculate light interception for canopy layers. The proposed 3D model provides a pivotal tool in characterizing the light interception by the individual organ and even its facets, allowing a more accurate description of canopy photosynthetic production.

### Potential limitations

The 3D model for calculating maize yield potential assumed that the grain yield is the accumulation of photosynthetic production without considering allocation and remobilization. This absence led to the aggravated discrepancy between yield potential and attainable yield at lower densities. Canopy photosynthesis module plays a key role in crop yield simulation using 1D, 2D, and 3D models which diverge in the approach to simulating light distribution and to upscaling in space. Despite a satisfactory agreement between simulation and observation in light distribution (Figure 4), the model performance in simulating canopy photosynthesis rate was not evaluated yet because of the limitation in measuring canopy-scale CO_2_ fluxes for tall plants. Accuracy and efficiency are always a trade-off in crop modeling. Acquiring 3D digitized point data for 3D canopy reconstruction in this study is time-consuming, but an increasing amount of high-throughput 3D phenotyping technology and algorithm become available to overcome this limitation (Wang et al., 2018). The software we used in this study is also compatible with the point data generated by the 3D laser scanning and multi-view stereo reconstruction (Wang et al., 2018), facilitating the combination of flexibility and accuracy in our model in the future study. Organ development, water utilization, and leaf nitrogen in 3D space are involved in canopy photosynthetic production and have been included in different 3D maize canopy models such as ADEL-Maize (Fournier and Andrieu, 1999), GREENLAB-Maize (Guo et al., 2006), and GRAAL (Drouet and Pagès, 2003). These factors need to be incorporated into the 3D model to explore complex interactions underlying canopy photosynthesis. For example, we assumed a uniform photosynthetic capacity for leaves within the upper, middle, and lower canopy layers for 1D, 2D, and 3D models for simplification (Supplementary Table 3). The absence of fully parameterization on leaf photosynthesis response curve over the entire canopy may result in departure from actual canopy photosynthesis. In addition, the 3D light model in this study simulated direct and diffuse light separately but did not consider the light reflection and transmission within canopy, which is closely related to leaf optical properties, and this may reduce the accuracy of simulating canopy photosynthesis. Hence, to what extent and how does the resolution of characterizing leaf photosynthetic capacity and leaf optical property affect canopy photosynthetic production and grain yield potential will be addressed in the future.

The difference in photosynthetic production between cultivars results from the integration of phenotypic traits across organizational levels from individual organs to entire canopies, whereas the contribution of each individual trait to canopy photosynthesis and yield formation remains unclear. To advance our knowledge on the function of primary phenotypic traits in determining sophisticated traits at the canopy level, canopy photosynthesis combined with 3D phenotyping techniques and 3D radiation model could be a promising approach.

## Data availability statement

The raw data supporting the conclusions of this article will be made available by the authors, without undue reservation.

## Author contributions

XG and CZ conceived and supervised the project and agreed to serve as the author responsible for contact and ensure communication. SG and WW developed the models. SG, TX, XL, and ZY conducted the experiment and collected the data. SG analyzed the data, prepared the figures, and wrote the manuscript. All authors contributed to the article and approved the submitted version.

## Footnotes

1
http://data.cma.cn/
