# Supplementary material for: Use of 3D modeling to refine predictions of canopy light utilization: A comparative study on canopy photosynthesis models with different dimensions
Source: Front Plant Sci. 2022 Aug 18;13:735981. doi: 10.3389/fpls.2022.735981 (PMC9434122; doi:10.3389/fpls.2022.735981)
Supplement: Supplementary file 2 [file Data_Sheet_1.docx]

**Supplementary file A**

*Crop parameters and environmental input module*

Crop parameters including canopy architectural traits, leaf photosynthetic capacity, and time of silking and maturity were included in the model. In the 0D, 1D and 2D model, LAI and *k* derived from the 3D model were set as input parameters to keep consistency, and daily total incoming PAR was the environmental input for calculating PAR interception by the canopy. The daily total incoming PAR and hourly incoming PAR intensity were set as the environmental input for the 0D and 1D model. In the 2D and 3D model, hourly incoming direct and diffuse PAR intensity were set as the environmental input for simulating light distribution in canopy.

Incoming PAR in daily and hourly interval were calculated following the equations provided by Spitters et al. (1986) and Goudriaan and Laar (1994). The atmospheric transmissivity (*τ*) was calculated from the sunshine duration hours (*n*) and day length (D*_l_*) in the following form:

$$\tau=a+b(n/D_{l})$$

where a and b are empirical coefficients (0.17 and 0.53 calibrated by Ren et al. (2013) for the North China where the experimental site of this study belongs to). The fraction of diffuse radiation (*f_d_*) was calculated using the function of *τ* in SUCROS (Goudriaan and Laar, 1994) as equation S2.

$$f_{d}=\left\{\begin{matrix} 1\text{ }\tau\leq0.22 \\ 1-6.4(\tau-0.22)^{2}\text{ 0.22<}\tau\leq0.35 \\ 1.47-1.66\tau\text{ }\tau>0.35 \end{matrix}\right.$$

*3D canopy photosynthesis model*

The instantaneous net photosynthesis rate of leaf at the *i*th rank of the *j*th plant within the focal area of constructed canopy at the time *t* (*A_i,j,t_* μmol m^-2^ s^-1^) was calculated as:

$$A_{i,j,t}\text{=}\frac{\alpha I_{i,j,t}+A_{\max,i}-\sqrt{\left(\alpha I_{i,j,t}+A_{\max,i}\right)^{2}-4\theta\alpha I_{i,j,t}A_{\max,i}}}{2\theta}$$

where *I_i_*_,_*_j_*_,_*_t_* is the average incident PAR intensity for the leaf at rank *i* of the *j*th plant during the hour of *t*, *A*_max,_*_i_* is the net photosynthesis rate at the saturated PAR intensity for the leaf at rank *i*, *α* is the apparent quantum yield, *θ* is the an empirical coefficient determining the curvature.

The *A_i,j,t_* was then summed to obtain instantaneous canopy CO_2_ assimilation (*A*_can,_*_t_*) in the following forms:

where *A_plant,j_* (μmol plant^-1^ s^-1^) is the instantaneous CO_2_ assimilation of the entire plant *j*, *L_i,j_* the leaf area of the leaf *i* in the plant *j*, *N_j_* the total number of leaves of the plant *j*, *M* the total number of plants in the focal area of constructed canopy, *D* the plant density (plants m^-2^).

After integrating *A*_can,_*_t_* to the daily scale, daily assimilated CO_2_ by the canopy (*A*_canDAY,_*_d_*) can be obtained by the following equation.

$$A_{\text{canDAY,}d}\text{=3600}\sum_{t=sunrise}^{sunset} A_{\text{can},t}$$

The daily intercepted PAR by the canopy on the day *d* of year was calculated as:

where *IPARplt*_DAY_*_,j,d_* is the intercepted PAR by the plant *j* in the focal canopy on the day *d* of year, and the conversion factor of 4.55 μmol J^-1^ was used to convert PAR in μmol m^-2^ s^-1^ to PAR in J m^-2^ s^-1^ over the canopy (Goudriaan and Laar, 1994). This conversion factor was assumed to be constant over the canopy regardless of the spectral distribution inside the canopy.

*2D canopy photosynthesis model*

The LAI of the canopy was equally divided into 10 layers with an interval of LAI/10, each of which was decomposed into sunlit and shaded leaves. The extinction coefficient for direct PAR for black leaves was expressed as:

$$K\text{dir}_{t}=\frac{O_{\text{av}}}{\sin\beta_{t}}$$

where *O*_av_ is the average projection of leaves in the direction of a solar beam and *β_t_* the solar elevation at the hour *t* of the day. By assuming that the leaves in a canopy have a uniform azimuth orientation, *O*_av_ can be calculated as:

$$O_{\text{av,}a}=\left\{\begin{matrix} \sin\beta_{t}\cos\beta_{La}\text{ }\beta_{t}\geq\beta_{La} \\ \frac{2}{\pi}\left[\text{sin}\beta_{t}\cos\beta_{\text{La}}\arcsin\left(\frac{\tan\beta_{t}}{\tan\beta_{La}}\right)+\sqrt{\text{sin}^{2}\beta_{La}-\text{sin}^{2}\beta_{t}}\right]\text{ }\beta_{t}<\beta_{La} \end{matrix}\right.$$

where *β_La_* is the leaf inclination angle. For a canopy with nine 10° inclination classes the *O*_av_ can be calculated as:

$$O_{\text{av}}\text{= }\sum_{a=1}^{9} f_{a}O_{\text{av},a}$$

where *f_a_* is the fraction of the leaves in the *a*th leaf inclination class.

The direct (*I*dir*_n,t_*) and diffuse (*I*diff*_n,t_*) incident PAR per unit ground area at the top of the *n*th canopy layer at the hour *t* of the day was calculated as:

where *K*diff is the extinction coefficient for diffuse PAR.

The intercepted PAR by the entire canopy at the hour *t* of the day was calculated as:

$$IPAR_{t}\text{=}\sum_{n=1}^{10} \left(\Delta I\text{dir}_{n,t}\text{+}\Delta I\text{diff}_{n,t}\right)$$

where (∆*I*dir*_n,t_*) is direct and (∆*I*diff*_n,t_*) is diffuse PAR by the *n*th layer of canopy that was calculated as:

where *I*dir*_n,t_* and *I*diff*_n,t_* were expressed in S8 and S9.

The daily intercepted PAR by the entire canopy at the day *d* of year (*IPAR*_DAY_*_,d_*) in MJ m^-2^ d^-1^ was calculated as:

$$IPAR_{\text{DAY},d}\text{=}\frac{\sum_{t=sunrise}^{sunset} 3600IPAR_{t}}{4.55}10^{-6}$$

where the conversion factor of 4.55 μmol J^-1^ was used to convert PAR in μmol m^-2^ s^-1^ to PAR in J m^-2^ s^-1^ over the canopy (Goudriaan and Laar, 1994).

The fraction of leaves that are sunlit (*f*sun*_n,t_*) and shaded (*f*sh*_n,t_*) in the *n*th canopy layer at the hour *t* of the day was calculated by the equation S27 and S28.

The instantaneous photosynthesis rate for sunlit leaves (*Asun_n,t_*) and shaded leaves (*Ash_n,t_*) in the nth canopy layer was calculated by the non-rectangular hyperbola function:

where *α* indicates apparent quantum yield, *θ* the empirical coefficient, *I*sun*_n,a,t_* the total intercepted PAR per unit leaf area by sunlit leaves in the *a*th leaf angle class in the *n*th canopy layer at the hour *t* of the day, and *I*sh*_n,t_* the total intercepted PAR per unit leaf area by shaded leaves in the *n*th canopy layer at the hour *t* of the day. *I*sun*_n,a,t_* and *I*sh*_n,t_* can be calculated as:

where *ξ_a,t_* indicates leaf inclination with respect to direct radiation.

The total intercepted PAR per unit leaf area in the *n*th canopy layer can be expressed using the following equation S33.

Instantaneous canopy photosynthesis rate at the hour *t* of the day (*A*_can_*_,t_*) can be calculated by the following equation S34.

$$A_{\text{can},t}=\sum_{n=1}^{10} \frac{LAI}{10}\left\{\left[fsun_{n,t}\left(\sum_{a=1}^{9} f_{a}Asun_{n,a,t}\right)\right]+fsh_{n,t}Ash_{n,t}\right\}$$

After integrating *A*_can,_*_t_* to the daily scale, daily assimilated CO_2_ by the canopy (*A*_canDAY,_*_d_*) can be obtained by the equation S6.

*1D canopy photosynthesis model*

A three-point Gaussian integration was applied to calculate canopy photosynthesis (Goudriaan, 1986). Three canopy depths were selected symmetrically as:

where LAI is the total leaf area index, *L*_1_ is the upper level, *L*_2_ is the middle level, and *L*_3_ is the lower level of the canopy. The absorbed PAR at these three levels were expressed as:

where *k* is the light extinction coefficient, *I*_0,_*_t_* is the incident PAR above canopy at the hour *t* of day. The corresponding instantaneous photosynthesis rate (*A*_1,_*_t_*, *A*_2,_*_t_*, and *A*_3,_*_t_*) were calculated as:

where *α* indicates apparent quantum yield, *θ* the empirical coefficient, *A*_max,1_, *A*_max,2_, *A*_max,3_ is the net photosynthesis rate at the saturated PAR intensity at the upper, middle, and lower canopy level.

Instantaneous canopy photosynthesis rate at the hour *t* of the day (*A*_can_*_,t_*) can be calculated by the following equation.

$$A_{\text{can},t}\text{=}\left(0.277778A_{1,t}+0.444444A_{2,t}+0.277778A_{3,t}\right)LAI$$

Daily assimilated CO_2_ by the canopy (*A*_canDAY,_*_d_*) can be obtained using the equation S6.

The intercepted PAR at the hour *t* of the day (*IPAR_t_*) was calculated by the following equation.

$$IPAR_{t}\text{=}I_{0,t}\left(1-e^{-kLAI}\right)$$

The daily intercepted PAR by the entire canopy at the day *d* of year (*IPAR*_DAY_*_,d_*) was calculated by the equation S17.

*0D canopy photosynthesis model*

Given the daily total incoming PAR (*I*_0_*_,d_*), leaf area index of the canopy (LAI), and light extinction coefficient (*k*), the daily dry mass production of canopy (*DM_d_*) was calculated by equations S3-S4:

where RUE is the radiation use efficiency and *fIPAR* the fraction of intercepted PAR by canopy.

*Yield formation module*

The daily canopy dry mass increment (*DM_d_*) can be calculated by:

$$DM_{d}=44A_{\text{canDAY},d}C_{\text{r}}$$

where 44 is mole mass of CO_2_ and *C*_r_ is the conversion efficiency from CO_2_ to dry mass in maize.

The daily RUE was accordingly calculated as:

$$RUE_{\text{DAY,}d}=\frac{DM_{d}}{IPAR_{\text{DAY},d}}$$

The grain yield potential (*Y_p_*) was computed by summing the *DM_d_* over the post-silking period using the equation S42.

$$Y_{p}=\sum_{d=DOY_{\text{silking}}}^{DOY_{\text{maturity}}} DM_{d}$$

Table S1 Description of symbols used in the 0D, 1D, 2D, and 3D model

| **Symbol** | **Description** | **Units** | **Models** | **Value** | **Source** | **Equation** |
| --- | --- | --- | --- | --- | --- | --- |
| **Crop parameters and environmental input module** | | | | | | |
| LAI | Leaf area index | m^2^ m^-2^ | 0D, 1D, 2D |  | This study | S26, S27, S28, S35, S36, S38 |
| *k* | Light extinction coefficient |  | 0D, 1D |  | This study | S29, S30, S31, S36 |
| RUE | Radiation use efficiency during post-silking period | g MJ^-1^ | 0D | 2.5 | (Zhao et al., 2015) | S37 |
| *f_a_* | Fraction of the leaves in the leaf inclination class *a* |  | 2D | Table S2 | Table S2 of this study | S11 |
| *β_La_* | Mean leaf inclination in the class *a* | ° | 2D | 5, 15, 25, 35, 45, 55, 65, 75, 85 | Table S2 of this study | S10 |
| *L_i,j_* | Leaf area of the leaf *i* of the plant *j* | m^2^ | 3D |  | This study | S4 |
| *N_j_* | Total number of leaves of the plant *j* |  | 3D | 200 | This study | S4 |
| DOY_silking_ | Day of year at silking | d | 0D, 1D, 2D, 3D | 260 | This study | S42 |
| DOY_maturity_ | Day of year at maturity | d | 0D, 1D, 2D, 3D |  | This study | S42 |
| DAS | Days after silking | d | 1D, 2D, 3D |  | This study | Table S3 |
| *A*_max,_*_n_*  *A*_max,_*_i_* | Photosynthesis rate at saturated light conditions in the layer *n* of canopy and for the leaf *i* | μmol CO_2_ m^2^ s^-1^ | 1D, 2D, 3D | Table S3 | (Chen et al., 2016) | S3, S20, S21, S32, S33, S34 |
| *α* | Apparent quantum yield |  | 1D, 2D, 3D | 0.05 | This study | S3, S20, S21, S32, S33, S34 |
| *θ* | Empirical curvature coefficient |  | 1D, 2D, 3D | 0.8 | This study | S3, S20, S21, S32, S33, S34 |
| *I*_0,d_ | Daily total incoming PAR | MJ m^-2^ d^-1^ | 0D |  | (Goudriaan and Laar, 1994) | S39 |
| *τ* | Atmospheric transmissivity |  | 0D, 1D, 2D, 3D |  | (Ren et al., 2013) | S1, S2 |
| *n* | Sunshine duration hours | h | 0D, 1D, 2D, 3D |  | This study | S1 |
| *D_l_* | Day length | h | 0D, 1D, 2D, 3D |  | This study | S1 |
| *a, b* | Empirical coefficients |  | 0D, 1D, 2D, 3D | 0.17, 0.53 | (Ren et al., 2013) | S1 |
| *f_d_* | Fraction of diffuse radiation |  | 2D, 3D |  | (Goudriaan and Laar, 1994) | S2 |
| *I*_0,t_ | Total incident PAR at the hour *t* of day | μmol photons m^2^ s^-1^ | 1D |  |  | S29, S30, S31 |
| *I*dir_0,_*_t_* | Direct incident PAR at the hour *t* of day | μmol photons m^2^ s^-1^ | 2D, 3D |  |  | S12 |
| *I*diff_0,_*_t_* | Diffuse incident PAR at the hour *t* of day | μmol photons m^2^ s^-1^ | 2D, 3D |  |  | S13 |
| **Canopy photosynthesis module** | | | | | | |
| *fIPAR* | Fraction of intercepted PAR |  | 0D, 1D |  |  | S39 |
| *IPAR*_DAY_*_,d_* | Intercepted PAR by the canopy on the *d* day of year | MJ m^-2^ d^-1^ | 0D, 1D, 2D, 3D |  |  | S8, S17, S39 |
| *IPARplt*_DAY_*_,j,d_* | Intercepted PAR by the plant *j* in the focal canopy on the day *d* of year | MJ plant^-1^ d^-1^ | 3D |  |  | S7 |
| *IPAR_t_* | Intercepted PAR by the canopy foliage at the hour *t* of the day | μmol photons m^-2^ leaf s^-1^ | 2D |  |  | S14 |
| *K*dir*_t_* | Extinction coefficient for direct PAR for black leaves at the hour *t* of the day |  | 2D |  | (Goudriaan, 1988) | S12 |
| *K*diff | Extinction coefficient for diffuse PAR for black leaves |  | 2D | 0.7 | (Goudriaan, 2016) | S13 |
| *L_1_*, *L_2_*, *L_3_* | Cumulative leaf area index at the upper, middle, and lower canopy level | m^2^ m^-2^ | 1D |  | (Goudriaan, 1986) | S26, S27, S28 |
| *I_1_*, *I_2_*, *I_3_* | Absorbed PAR at the upper, middle, lower canopy level with a cumulative LAI of *L_1_*, *L_2_*, and *L_3_* | μmol photons m^-2^ leaf s^-1^ | 1D |  | (Goudriaan, 1986) | S29, S30, S31 |
| *A*_1,_*_t_*, *A*_2,_*_t_*, *A*_3,_*_t_* | Instantaneous photosynthesis rate at the upper, middle, lower canopy level with a cumulative LAI of *L_1_*, *L_2_*, and *L_3_* | μmol CO_2_ m^-2^ leaf s^-1^ | 1D |  | This study | S32, S33, S34 |
| *Ld* | Cumulative leaf area index at the canopy depth of *d* cm | m^2^ m^-2^ | 0D, 1D |  | This study | Eqn. 1 |
| *L_n_* | Cumulative leaf area index at the canopy layer of *n* | m^2^ m^-2^ | 2D |  | (Bonelli and Andrade, 2020) | S12, S13 |
| *I*dir*_n,t_* | Direct incident PAR per unit ground area at the top of the canopy layer *n* at the hour *t* of the day | μmol photons m^-2^ ground s^-1^ | 2D |  | (Bonelli and Andrade, 2020) | S15 |
| *I*diff*_n,t_* | Diffuse incident PAR per unit ground area at the top of the canopy layer *n* at the hour *t* of the day | μmol photons m^-2^ ground s^-1^ | 2D |  | (Bonelli and Andrade, 2020) | S16 |
| *I*sun*_n,a,t_* | Total intercepted PAR per unit leaf area by sunlit leaves of the leaf angle class *a* in the canopy layer *n* at the hour *t* of the day | μmol photons m^-2^ leaf s^-1^ | 2D |  | (Bonelli and Andrade, 2020) | S20 |
| *I*sh*_n,t_* | Total intercepted PAR per unit leaf area by shaded leaves in the canopy layer *n* at the hour *t* of the day | μmol photons m^-2^ leaf s^-1^ | 2D |  | (Bonelli and Andrade, 2020) | S23 |
| *I*leaf*_n,t_* | The total intercepted PAR per unit leaf area in the *n*th canopy layer | μmol photons m^-2^ leaf s^-1^ | 2D |  |  | S24 |
| *O*_av_ | Average projection of leaves in the direction of a solar beam |  | 2D |  | (Goudriaan, 1988) | S11 |
| *O*_av,_*_a_* | Projection of leaves with the leaf inclination class *a* in the direction of a solar beam |  | 2D |  |  | S11 |
| *ξ_a,t_* | Leaf inclination with respect to direct radiation | ° | 2D |  |  | S22 |
| *β_t_* | Solar elevation at the hour *t* of the day | ° | 2D |  |  | S22 |
| *fsun*_n,t_  *fsh*_n,t_ | Fraction of leaves that are sunlit and shaded in the canopy layer *n* at the hour *t* of the day |  | 2D, 3D |  | (Bonelli and Andrade, 2020) | S18, S19 |
| *Asun_n,t_*  *Ash_n,t_* | Instantaneous photosynthesis rate for leaves that are sunlit and shaded in the canopy layer *n* at the hour *t* of the day | μmol CO_2_ m^-2^ leaf s^-1^ | 2D, 3D |  | (Bonelli and Andrade, 2020) | S20, S21 |
| *I_i,j,t_* | Average total incident PAR intensity per leaf area for the leaf *i* of the plant *j* within the focal area of a reconstructed canopy at the time *t* of day | μmol photons m^-2^ leaf s^-1^ | 3D |  | This study | S3, S7 |
| *fsun_i,j,t_* | Fraction of facets that are sunlit for the leaf *i* of the plant *j* within the focal area of constructed canopy at the time *t* of day |  | 3D |  | This study | Fig. 9 |
| *A_i,j,t_* | Instantaneous net photosynthesis rate of leaf *i* of the plant *j* within the focal area of a reconstructed canopy at the time *t* | μmol CO_2_ m^-2^ leaf s^-1^ | 3D |  | This study | S3 |
| *A*_plant_*_,j_* | Instantaneous CO_2_ assimilation of the entire plant *j* | μmol CO_2_ plant^-1^ s^-1^ | 3D |  | This study | S4 |
| *A*_can_*_,t_* | Canopy photosynthesis rate at the hour *t* of the day | μmol CO_2_ m^-2^ s^-1^ | 1D, 2D, 3D |  |  | S5, S25, S35 |
| *A*_canDAY,_*_d_* | Daily assimilated CO_2_ by the canopy on the day *d* of year | μmol CO_2_ m^-2^ d^-1^ | 1D, 2D, 3D |  |  | S6 |
| **Yield formation module** | | | | | | |
| *C*_r_ | Conversion coefficient from CO2 to dry mass | g g^-1^ | 1D, 2D, 3D | 0.41 | (Sinclair and Horie, 1989) | S40 |
| *DM_d_* | Daily dry mass of canopy on the *d* day of year | g m^-2^ d^-1^ | 0D, 1D, 2D, 3D |  |  | S40, S41, S42 |
| *CDM* | Cumulative dry mass of canopy | g m^-2^ | 0D, 1D, 2D, 3D |  |  | Fig. 6 |
| *CIPAR* | Cumulative intercepted PAR by the canopy | MJ m^-2^ | 0D, 1D, 2D, 3D |  |  | Fig. 6 |
| *RUE*_DAY,_*_d_* | Daily radiation use efficiency on the *d* day of year | g MJ^-1^ | 1D, 2D, 3D | - |  | S41 |
| *Y*_p_ | Predicted grain yield | Mg ha^-1^ | 1D, 2D, 3D | - |  | S42 |

Table S2 Leaf inclination distribution *f_a_* for 10° class width calculated by the relative frequency across phytomers based on randomly selected 10 plants in each treatment

| Leaf inclination class | XY4.5 | XY7.5 | XY10.5 | ZD7.5 |
| --- | --- | --- | --- | --- |
| 0-10° | 0 | 0 | 0 | 0 |
| 10-20° | 0 | 0 | 0 | 0 |
| 20-30° | 0 | 0 | 0 | 0 |
| 30-40° | 0 | 0 | 0 | 0 |
| 40-50° | 0 | 0 | 0.009 | 0 |
| 50-60° | 0.154 | 0.230 | 0.090 | 0.081 |
| 60-70° | 0.769 | 0.770 | 0.802 | 0.718 |
| 70-80° | 0.077 | 0 | 0.099 | 0.201 |
| 80-90° | 0 | 0 | 0 | 0 |

Table S3 The *A*_max_ for simulation using the 1D, 2D, and 3D with and without considering spatial-temporal pattern of photosynthetic capacity

| 1D model | 2D model | 3D model | *A*_max_ (μmol m^2^ s^-1^) | |
| --- | --- | --- | --- | --- |
|  |  |  | Dynamic and heterogeneous | Constant and homogenous |
| 0.8873LAI | 8-10th layers | Rank ≤ 11 | *A*_max_ = -0.39DAS+21.88 | 37.82 |
| 0.5LAI | 4-7th layers | 11 < Rank ≤ 16 | *A*_max_ = -0.44DAS+32.93 | 37.82 |
| 0.1127LAI | 1-3th layers | rank > 16 | *A*_max_ = -0.49DAS+37.82 | 37.82 |

Note: the factors before LAI indicate the canopy depths and the DAS indicates days after silking.

Table S4 Simulation scenarios for evaluating the effects of high-resolution canopy photosynthesis model on maize yield potential

| Scenario | 0D model | 1D model | 2D model | 3D model | Uniform and constant RUE or *A*_max_ | Heterogenous and varied *A*_max_ |
| --- | --- | --- | --- | --- | --- | --- |
| 0D_constant | ● | ○ | ○ | ○ | ● | ○ |
| 1D_constant | ○ | ● | ○ | ○ | ● | ○ |
| 2D_constant | ○ | ○ | ● | ○ | ● | ○ |
| 3D_constant | ○ | ○ | ○ | ● | ● | ○ |
| 1D_varied | ○ | ● | ○ | ○ | ○ | ● |
| 2D_vaired | ○ | ○ | ● | ○ | ○ | ● |
| 3D_varied | ○ | ○ | ○ | ● | ○ | ● |

Closed circles indicate the module is included, and open circles indicate the module is not included.


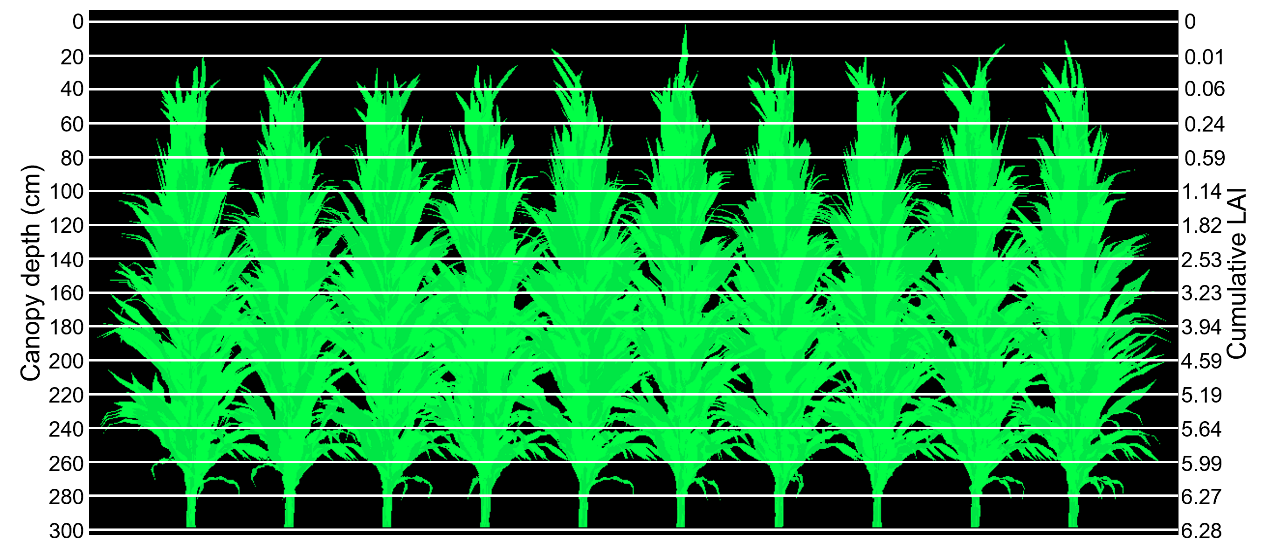


Figure S1 The cumulative LAI at different canopy depths for the XY335 at the density of 7.5 plants m^-2^ generated by the 3D canopy architecture module “MaizeTypeOpt”


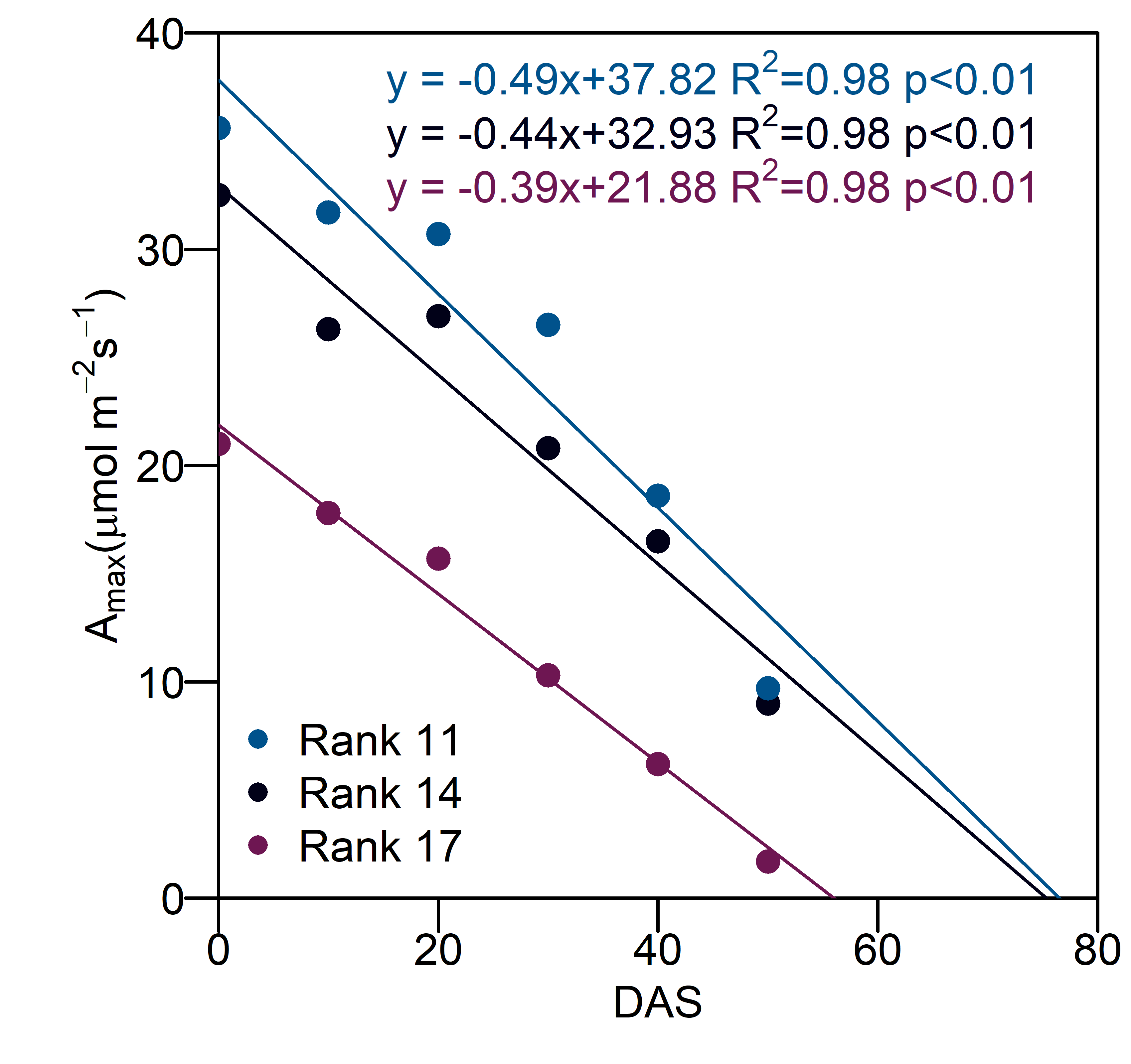


Figure S2 The relationship between *A*_max_ and DAS for leaves at the rank 11, 14, and 17 for the cultivar of XY335


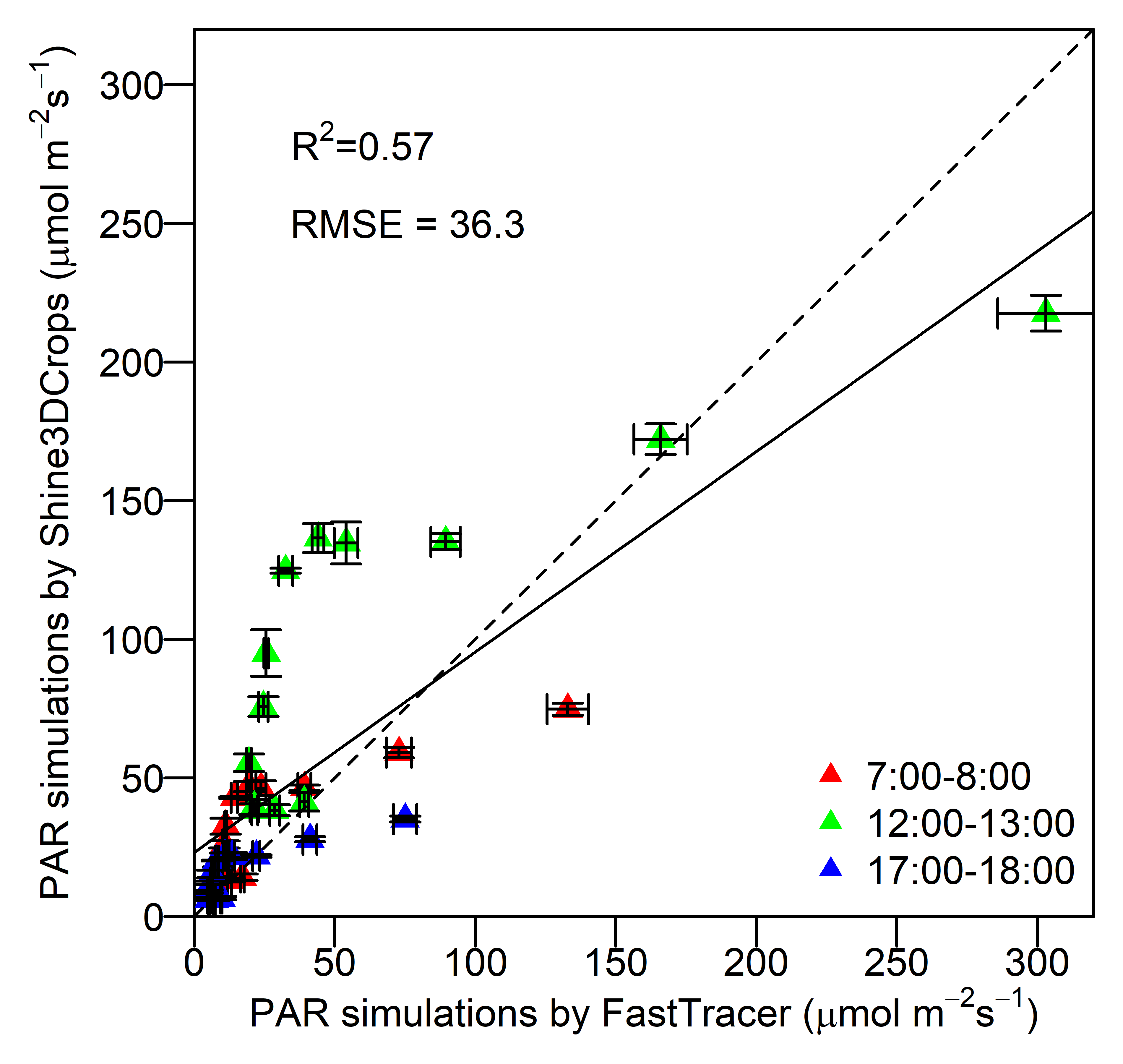


Figure S3 The comparison between PAR interception by individual leaves simulated by the Shine3DCrops and that by the FastTracer for the cultivar of XY335 at 7.5 plants m^-2^ on September 3 2021. Red triangles represent average PAR interception intensity during 7:00-8:00, green represent that during 12:00-13:00, and blue represent that during 17:00-18:00.

**Bonelli LE, Andrade FH** (2020) Maize radiation use-efficiency response to optimally distributed foliar-nitrogen-content depends on canopy leaf-area index. Field Crops Research **247:** 107557

**Chen Y, Wu D, Mu X, Xiao C, Chen F, Yuan L, Mi G** (2016) Vertical Distribution of Photosynthetic Nitrogen Use Efficiency and Its Response to Nitrogen in Field-Grown Maize. Crop Science **56:** 397-407

**Goudriaan J** (1986) A simple and fast numerical method for the computation of daily totals of crop photosynthesis. Agricultural and Forest Meteorology **38:** 249-254

**Goudriaan J** (1988) The bare bones of leaf-angle distribution in radiation models for canopy photosynthesis and energy exchange. Agricultural and Forest Meteorology **43:** 155-169

**Goudriaan J** (2016) Light Distribution. *In* K Hikosaka, Ü Niinemets, NPR Anten, eds, Canopy Photosynthesis: From Basics to Applications. Springer Netherlands, Dordrecht, pp 3-22

**Goudriaan J, Laar HHv** (1994) Modelling Potential Crop Growth Processes Textbook with exercises,

**Ren XL, He HL, Zhang L, Zhou L, Yu GR, Fan JW** (2013) Spatiotemporal variability analysis of diffuse radiation in China during 1981-2010. Annales Geophysicae **31:** 277-289

**Sinclair TR, Horie T** (1989) Leaf Nitrogen, Photosynthesis, and Crop Radiation Use Efficiency: A Review. Crop Science **29:** 90-98

**Spitters CJT, Toussaint HAJM, Goudriaan J** (1986) Separating the Diffuse and Direct Component of Global Radiation and Its Implications for Modeling Canopy Photosynthesis .1. Components of Incoming Radiation. Agricultural and Forest Meteorology **38:** 217-229

**Zhao J, Yang X, Lin X, Sassenrath GF, Dai S, Lv S, Chen X, Chen F, Mi G** (2015) Radiation Interception and Use Efficiency Contributes to Higher Yields of Newer Maize Hybrids in Northeast China. **107:** 1473-1480
