# Supplementary material for: Use of 3D modeling to refine predictions of canopy light utilization: A comparative study on canopy photosynthesis models with different dimensions
Source: Front Plant Sci. 2022 Aug 18;13:735981. doi: 10.3389/fpls.2022.735981 (PMC9434122; doi:10.3389/fpls.2022.735981)

**Supplementary file B**

A tutorial demonstrating how to use the Shine3DCrops to calculate light distribution within a 3D canopy

**Platform and software**

Shine3DCrops is a software which supports calculation of radiation distribution based on a 3D crop canopy.

**Installation and requirements**

Shine3DCrops is a pure C++ application and releases pre-packaged versions for Windows currently. For Windows users, there is an installer package containing Shine3DCrops together with needed modules files. Just click Shine3DCrops.zip, decompress and run it.

**Screenshots of Shine3DCrops**

Shine3DCrops has five modules: 1) incident PAR analysis; 2) instantaneous PAR distribution; 3) accumulated PAR in a day; 4) accumulated PAR over a period; 5) batch processing tool.


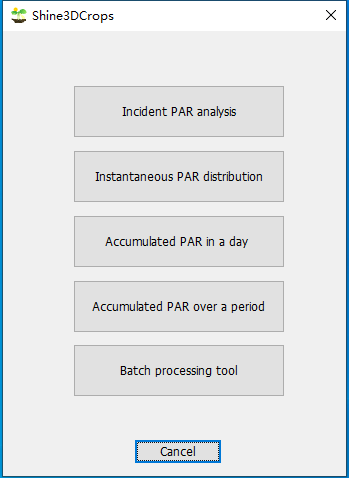


Fig. 1 The main interface of Shine3DCrops


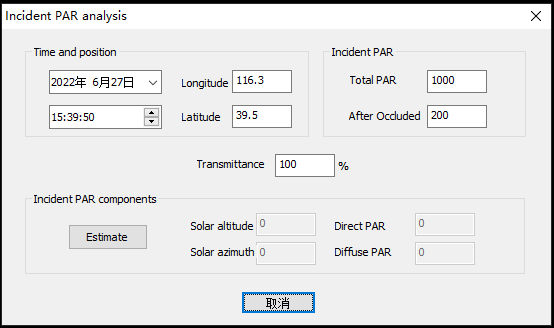


Fig. 2 The interface of incident PAR analysis


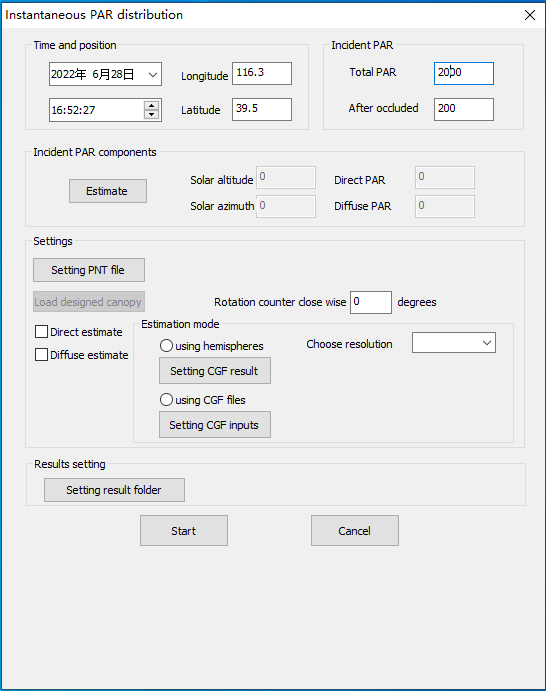


Fig. 3 The interface of instantaneous PAR distribution


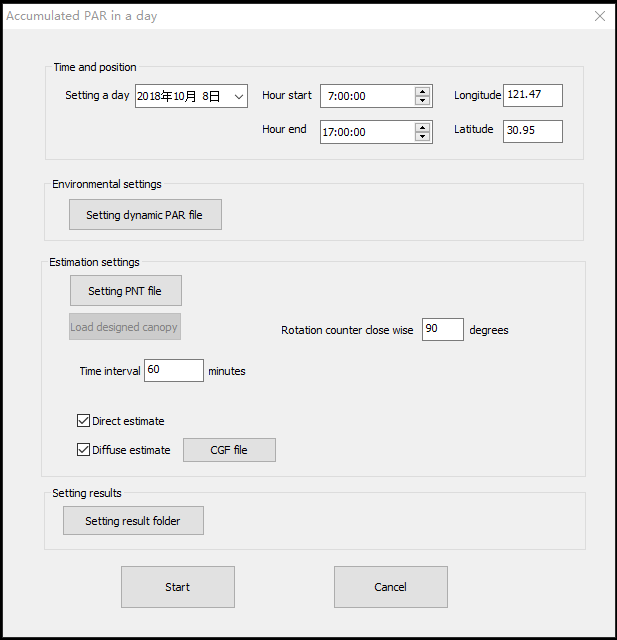


Fig. 4 The interface of accumulated PAR in a day


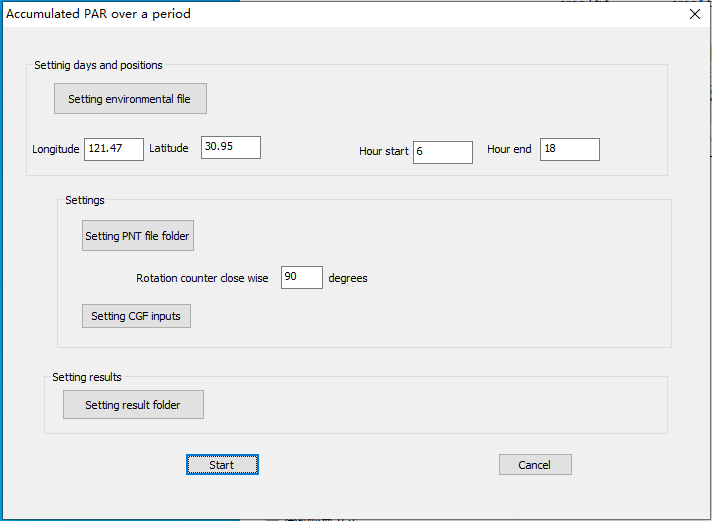


Fig. 5 The interface of accumulated PAR during a period


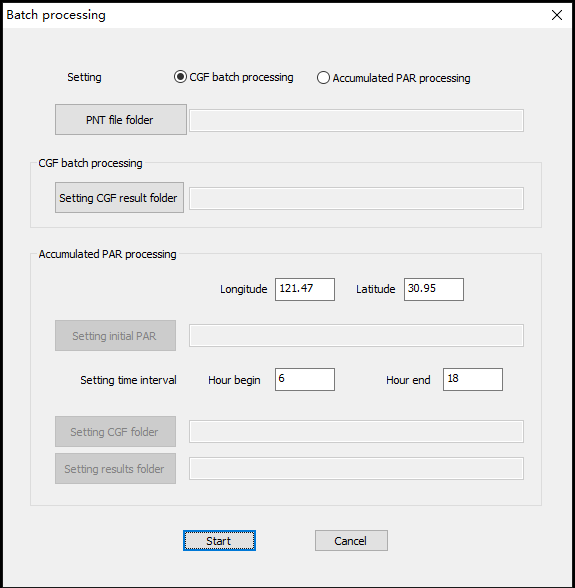


Fig. 6 The interface of batch processing

**Model input for the Shine3DCrops**

Two files need to be prepared for calculating PAR distribution. One is 3D reconstructed crop canopy. The other is climate input.

The 3D reconstructed maize canopy is consisting of several individual plants, and each plant has leaf, sheath, internode and cob. It is a point cloud data in the format of pnt. Each organ is composed of several facets and has some properties like plant ID and organ ID.


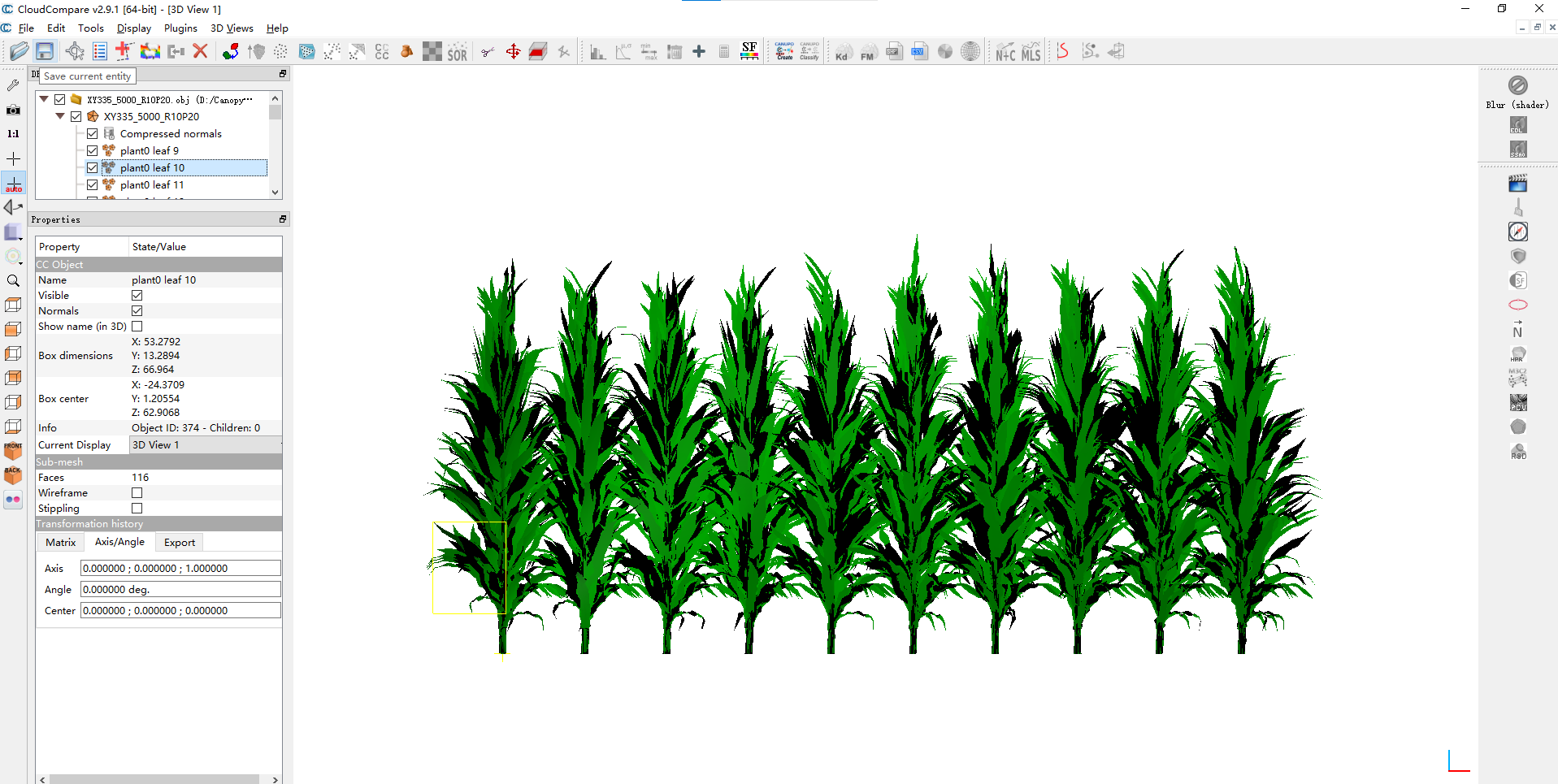


Fig. 7 The reconstructed 3D maize canopy viewed in the CloudCompare

The climate input file in the txt file includes longitude, latitude, day of year, hourly incident PAR intensity of direct and diffuse light.

**Intermediate file in Shine3DCrops**

Canopy gap fractions (CGFs) of each canopy, which contained the probability of a diffuse beam reaching to each facet in the canopy, were calculated first (Wen et al., 2019). It is a cgf file and required to be loaded when calculating PAR distribution for a 3D crop canopy. More details can be seen in Wen et al., 2019.

Wen W, Guo X, Li B, Wang C, Wang Y, Yu Z, Wu S, Fan J, Gu S, Lu X (2019) Estimating canopy gap fraction and diffuse light interception in 3D maize canopy using hierarchical hemispheres. Agricultural and Forest Meteorology 276-277: 107594

**Model output in Shine3DCrops**

In the case of calculating PAR over a certain period, the climate input file should be loaded via the setting the environmental file button and the longitude and latitude need to be filled as the experimental station. Then the 3D reconstructed canopy file and the cgf file need to be loaded in the corresponding settings. After setting the saving path via the button of setting result folder, users can click the start button to run the calculation. The output will be in the format below.


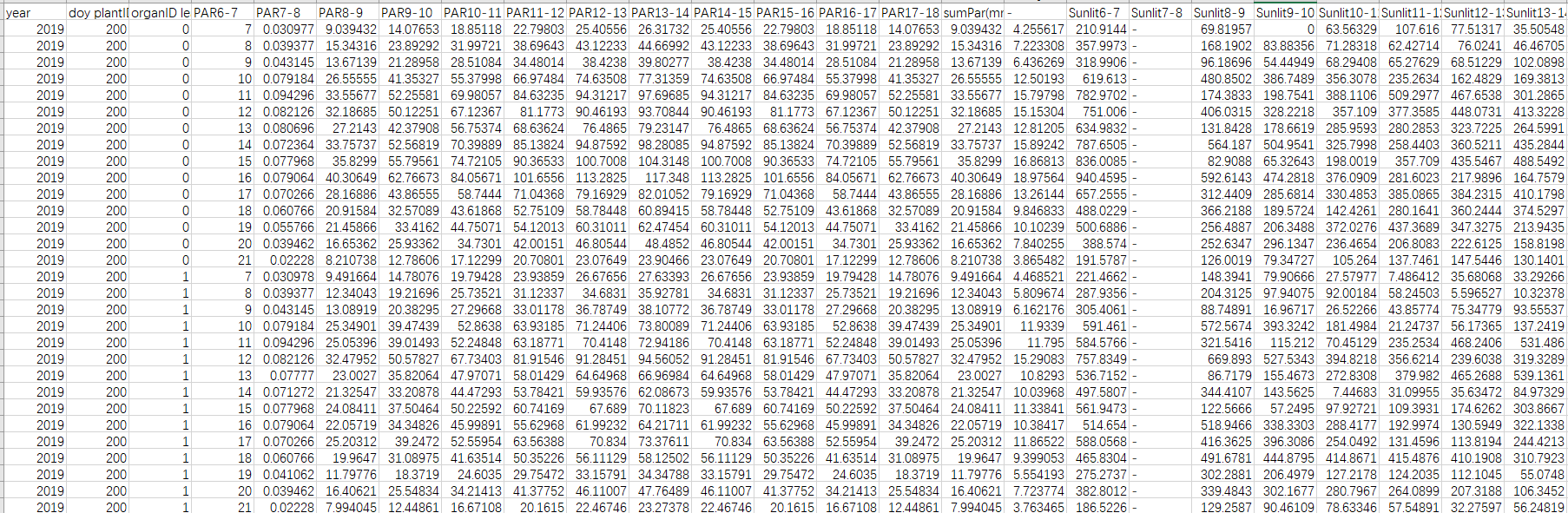

Supplement: Supplementary file 3 [file Data_Sheet_2.docx]
